# Supplementary material for: A restricted dynamic surface self-reconstruction toward high-performance of direct seawater oxidation
Source: Nat Commun. 2024 Mar 20;15:2481. doi: 10.1038/s41467-024-46708-8 (PMC10954752; doi:10.1038/s41467-024-46708-8)
Supplement: Supplementary file 1 — Supplementary Information [file 41467_2024_46708_MOESM1_ESM.pdf]

Supplementary Information

A restricted dynamic surface self-reconstruction toward high-performance of direct seawater oxidation

Ling Zhou,[1] Daying Guo,[1]* Lianhui Wu,[1] Zhixi Guan,[1] Chao Zou,[1] Huile Jin,[1] Guoyong Fang,[1] Xi'an Chen,[1]* and Shun Wang[1]*

[1] Key Laboratory of Carbon Materials of Zhejiang Province, College of Chemistry and Materials Engineering, Wenzhou University, Wenzhou, China 325035.

* To whom correspondence should be addressed. E-mail: guody@wzu.edu.cn; xianchen@wzu.edu.cn; shunwang@wzu.edu.cn

Table of Contents:

Experimental Section
Preparation of MoO_3-CoO/CC, MoO_3/CC and RuO_2/CC catalysts
Materials characterizations
Electrochemical measurements
Rotating ring-disk studies (RRDE) of chlorine evolution measurements
Gas chromatography measurement.
Chloride ion concentration test.
Electrochemical calculation.
Density functional theory methods.
X-ray absorption spectra (XAS) measurements
Supplementary Fig. 1. Morphology analysis of beaded-like Co(OH)F.
Supplementary Fig. 2. Morphology analysis of beaded-like CoO.
Supplementary Fig. 3. Morphology analysis of cowpea-like MoO_3@CoO/CC.
Supplementary Fig. 4. XPS analysis.
Supplementary Fig. 5. Polarization curve.
Supplementary Fig. 6. Electric double layer capacitance test.
Supplementary Fig. 7. Cyclic voltammetric curve testing of various samples.
Supplementary Fig. 8. Electrochemical active area (ESCA) normalization.
Supplementary Fig. 9. The turnover frequency calculation.
Supplementary Fig. 10. Density functional theory (DFT) calculation model.
Supplementary Fig. 11. The differential charge density after adsorption of various small molecules.
Supplementary Fig. 12. Morphology analysis of various samples after OER in seawater.
Supplementary Fig. 13. Electron energy loss spectrum analysis of MoO_3@CoO/CC-aft catalyst.
Supplementary Fig. 14. Measurement of electric double layer capacitance after seawater oxidation.
Supplementary Fig. 15. Structural analysis of various samples after OER in seawater.
Supplementary Fig. 16. X-ray absorption near edge structure (XANES) analysis.
Supplementary Fig. 17. K-space fitting structure model.
Supplementary Fig. 18. Wavelet transform (WT)-EXAFS analysis of Co K-edge.
Supplementary Fig. 19. Co-k edge FT-EXAFS fitting and k space fitting curves.
Supplementary Fig. 20. Mo-k edge FT-EXAFS fitting and k space fitting curves.
Supplementary Fig. 21. Wavelet transform (WT)-EXAFS analysis of Mo K-edge.
Supplementary Fig. 22. Schematic diagram of structural transformation of various samples.
Supplementary Fig. 23. Diffusion dynamics analysis.
Supplementary Fig. 24. DFT calculation model.
Supplementary Fig. 25. DFT calculation adsorption model.
Supplementary Fig. 26. The differential charge density.
Supplementary Fig. 27. Schematic diagram of the catalytic mechanism.
Supplementary Fig. 28. The influence of TMAOH for various catalysts.
Supplementary Fig. 29. Analysis of chlorine evolution and oxygen production efficiency of catalysts in various electrolytes.
Supplementary Fig. 30. Performance of two-electrode system.
Supplementary Fig. 31. Characteristics analysis of flow electrolytic cell.

Supplementary Table 1. AC impedance fitting.
Supplementary Table 2. Performance comparison.
Supplementary Table 3. Structural parameters extracted from the EXAFS fitting.
Supplementary Table 4. The free energy changes.
Supplementary Table 5. Performance comparison under different conditions.
Supplementary Table 6. Comparison of stability of continuous oxygen generation with literature.
Supplementary Table 7. Comparison of hydrogen production rate with literature.
References (S1-S49).

Experimental Section

Preparation of MoO_3-CoO/CC, MoO_3/CC and RuO_2/CC catalysts: To prepare different electrodes for comparison, the mixture of 1 mg MoO_3, 100 μL ethanol and 30 μL Nafion were ultrasonic treated for 30 min, then the dispersion was coated on CoO/CC substrates and air dried to obtain MoO_3-CoO/CC catalyst. Also, the mixture of 2 mg RuO_2, 100 μL ethanol and 30 μL Nafion were ultrasonic treated for 30 min, then the dispersion was coated on CoO/CC substrates and air dried to obtain RuO_2/CC catalyst. In addition, MoO_3/CC catalyst was prepared by ALD MoO_3 process on CC substrates for 500 cycles.

Materials characterizations: The nanostructure and morphology. of catalysts were characterized by scanning electron microscopy (Nova 200 Nano-SEM) and transmission electron microscopy (JEM-2100F) and the crystal structure was also analyzed by and X-ray powder diffraction (Bruker, D8 Advance) and Raman spectroscopy (in-Via, Renishaw). X-ray photoelectron spectroscopy (ESCA lab 250 analyzer, Al Kα radiation excitation source) was used to further analyze the catalysts of phase compositions.

Electrochemical measurements: The OER performance were measured by an electrochemical workstation (DH7001A) in the three-electrode system. As-prepared samples were as working electrodes, a platinum wire as the counter electrodes and a standard Hg/HgO electrode as the reference electrode. LSV curves of OER tests were collected from 0 to 1 V *vs* Hg/HgO with scan rate of 20 mV s^{-1} in a 1 M KOH+0.5 M NaCl solution (PH≈14). All the potentials were converted to the reversible hydrogen electrode (RHE) according to the reference electrode calibration $E_{RHE} = E_{Hg/HgO} + 0.059pH + 0.098$ (V). Note that all data are measured after cyclic voltammetry (CV) is activated. With different working electrode sizes of about 4 cm^{-2}, the catalyst was activated by cyclic voltammograms (CV) until a stable polarization curve for each test sample. Electrochemical impedance spectroscopy (EIS) measurements were performed

by applying an alternating current (AC) voltage from 0.1 Hz to 100 KHz with an amplitude of 5 mV at open circuit potential. Converted to a RHE according to test correction, the overpotential η was calculated according to formula $\eta = E_{RHE} - 1.23$ V.

Inductively Coupled Plasma Optical Emission Spectrometer（ICP-OES） measurements: The ICP-OES experiment, and calculated the contents of CoO and MoO_3 in MoO_3@CoO/CC as 1.87 mg/cm^2 and 0.02 mg/cm^2, respectively, according to the concentrations of Co^{2+} and Mo^{6+} in the sample.

Rotating ring-disk studies (RRDE) of chlorine evolution measurements: RRDE measurements were done with a MSR rotator and E6 Change Disk RRDE tips in a PEEK shroud (Pine Research). The RRDE test used a platinum-carbon electrode as the working electrode. The RRDE consisted of a glassy carbon with a diameter of 5.61 mm and a platinum ring with a diameter of 6.25 mm. MoO_3@CoO and CoO were ultrasonic removed from the carbon cloth, the mixture of 10 mg samples, 100 μL ethanol and 10 μL Nafion were ultrasonic treated for 30 min to make ink, and then dropped on the ring electrode. LSV was tested at a scan rate of 20 mV s^{-1} at 1500 rpm in alkaline electrolyte containing 30 mM chloride ions (PH=14). The ring was kept at 0.95 V during measurement of CER rates.

Gas chromatography measurement: OER tests were performed in a gas-tight electrochemical cell with 1 M KOH or 1 M KOH + 0.5/1/1.5/2 M NaCl electrolyte and SCE reference electrode. Chronopotientiometry was applied with different current densities to maintain constant oxygen generation. Meanwhile, Ar was constantly purged into the anodic compartment with a flow rate of 25 cm^3 min^{-1} and the compartment was connected to the gas-sampling loop of a gas chromatograph (SRI 8610C). A thermal conductivity detector (TCD) was used to detect and quantify the oxygen generated.

Chloride ion concentration test: The chloride concentration was measured using a LEICI ion meter (PXSJ-216F), 217-01 reference electrode and PCL-1-01 chloride ion electrode, measuring range (10^{-1}-10^{-5}) mol/L, error within 0.5%. The instrument was

calibrated with 0.001 mol L^{-1}, 0.01 mol L^{-1} and 0.1 mol L^{-1} NaCl solution before testing. In 1 M KOH+0.5 M NaCl solution, the it curves of the MoO_3@CoO/CC and CoO/CC were tested for 1 h, 10 h, 20 h and 50 h at the current density of 10 mA cm^{-2}, and the chloride ion concentration of the electrolyte after reaction at different times was measured respectively.

Electrochemical calculation: The electric double layer capacitance is calculated from the ratio of different current densities to different scanning rates at the same voltage:

$$C_{dl} = \frac{\Delta j}{v} \qquad (1)$$

$$\Delta j = \frac{j_a - j_c}{2} \qquad (2)$$

Where v is the different scanning rates, j_a and j_c are anode current and cathode current at a voltage of -0.056 V (*vs* RHE), respectively. Data were obtained from the cyclic voltammogram the region of -0.106 ~ -0.006 V *vs* RHE.

ECSA of working electrode is acquired form the following equation:

$$\mathrm{ECSA} = \frac{C_{dl}}{C_s}$$

Where C_s is the specific capacitance for a flat surface in the range of 60 μF cm^{-2} in basic solutions.

The turnover frequency (TOF) of OER is calculated by the equation.

$$TOF = \frac{j \times A}{4 \times F \times n}$$

Where j represents the current density (A cm^{-2}) at an overpotential of different potential, A and n represent the area of the electrode and the moles of the active materials, respectively. F is Faraday's constant (96485.3 C mol^{-1}).

Proton reaction order: The dependence of OER reaction kinetics on proton activity is evaluated by proton reaction order (ρ^{RHE}), and the formula is as follows:

$$\rho^{RHE} = \partial \log(j) = \partial pH$$

In this experiment, the pH value is 12.5 to 14. Log(j) is the logarithm of current density at 1.6 V *vs* RHE. The closer the ρRHE value is to 1, it shows that OER kinetics will strongly depend on pH. This is mainly due to the uncoordinated proton-electron transfer

in the OER process.

Differential electrochemical mass spectrometry (DEMS) test: Firstly, the working electrode was activated and labeled in 0.1 M KOH prepared with $H_2{}^{18}O$ isotope for 10 minutes. After that, the electrode labeled with ^{18}O was washed with $H_2{}^{16}O$. Finally, the CV method was used to measure the oxygen evolution in 0.1 M KOH $H_2{}^{16}O$ solution at a scanning rate of 5 mV s^{-1} in the range of 1.1-1.6 V vs RHE. Meanwhile, the produced gas products are continuously monitored by mass spectrometry. To clarify the OER process of LOM directly, the MoO_3@CoO/CC catalyst was labeled with ^{18}O isotope. Firstly, MoO_3@CoO/CC was electrochemically activated with $H_2{}^{18}O$ solution in 0.1 M KOH electrolyte. Then, electrolyzed with $H_2{}^{16}O$ solution after labeling, and the generated oxygen product was verified by DEMS. The activated MoO_3@CoO/CC catalyst has obvious periodic intensity of $^{18}O^{16}O$ peak (mass-to-charge ratio, m/z = 34), while $^{18}O^{18}O$ has no signal (m/z = 36). This result suggests that the MoO_3@CoO/CC catalyst mechanism that undergoes activation induced directed reconfiguration transforms into LOM.

Flow cell seawater electrolyzer measurement (FCEM): Catalysts were prepared MoO_3@CoO/CC as an anodic (loading 1.89 mg cm^{-2}), and 1.5 mg cm^{-2} of Pt/C coated on the cathode. As a control, it was used for comparison with 1.5 mg cm^{-2} RuO_2 coated on the anode and 1.5 mg cm^{-2} Pt black coated on the cathode. The active area was controlled at 9 cm^2 (3 cm×3 cm). For the FCEM test, the temperature was controlled at 60°C, and preheated seawater (Among them, the real seawater comes from Dongtou District, Wenzhou, Zhejiang, China, and is directly used after filtering to remove sediment) was kept flowing through the cell at 20 mL min^{-1}. Activation was carried out prior to the test with a prepolarization at 0.4 A cm^{-2} for1 h. The steady-state polarization curve was de-termined via the galvanostatic method, and each point was recorded until the cell voltage was stable. The stability test in FCEM ran at 1 A cm^{-2} for 500 h. The amphoteric ionic nafion membranes used in FCEM. The treatment methods of membrane: The membrane is used directly after being immersed in the corresponding

electrolyte for 24 hours.

Calculation of energy efficiency and conversion rate: The Faradaic efficiency (FE) of all the products were calculated based on their corresponding electron transfer per molecule oxidation using the following equations.

$$\mathrm{FE}(\%) = \frac{n_{\text{experimentally product}}}{\mathrm{n}_{\text{theoretically product}}} \times 100\% \qquad (1)$$

$$\mathrm{n}_{\text{theoretically product}} = \frac{\mathrm{Q}}{\mathrm{n} \times \mathrm{F}} \qquad (2)$$

The electricity consumption per m^3 of H_2 produced was calculated as follows:

$$\mathrm{W} = \frac{\mathrm{n} \times \mathrm{F} \times \mathrm{U} \times 1000}{3600 \times \mathrm{V}_m}$$

where n is the molarity of electrons transferred for product, U is the input voltage and V_m is the molar volume of the gas at standard atmospheric pressure (22.4 mol L^{-1}). F is Faraday's constant.

X-ray absorption spectra (XAS) measurements: The X-ray absorption near edge structure (XANES) spectra were measured at beamline BL12B of National Synchrotron Radiation Laboratory (NSRL) of China. Data reduction, data analysis, and Extended X-ray Absorption Fine Structure (EXAFS) fitting were performed and analyzed with the Athena and Artemis programs of the Demeter data analysis packages [S1, S2] that utilizes the FEFF6 program [S3] to fit the EXAFS data. The energy calibration of the sample was conducted through standard Co foil and Mo foil, which as a reference was simultaneously measured. A linear function was subtracted from the pre-edge region, then the edge jump was normalized using Athena software. The $\chi(k)$ data were isolated by subtracting a smooth, third-order polynomial approximating the absorption background of an isolated atom. The $k3$-weighted $\chi(k)$ data were Fourier transformed after applying a Kaiser-Bessel window function (Δk = 1.0). For EXAFS modeling, the global amplitude EXAFS (CN, R, σ^2 and ΔE_0) were obtained by nonlinear fitting, with least-squares refinement, of the EXAFS equation to the Fourier-transformed data in R-space, using Artemis software, EXAFS of the Co foil and Mo foil are fitted and the obtained amplitude reduction factor S_0^2 value (0.753 and 0.803) was set in the EXAFS

analysis to determine the coordination numbers (CNs) in the Co/Mo-O/Co/Mo scattering path in sample.

Density functional theory methods: We have employed the first-principles [S4, S5] to perform all density functional theory (DFT) calculations within the generalized gradient approximation (GGA) using the Perdew-Burke-Ernzerhof (PBE) [S6] formulation. We have chosen the projected augmented wave (PAW) potentials [S7, S8] to describe the ionic cores and take valence electrons into account using a plane wave basis set with a kinetic energy cutoff of 400 eV. Partial occupancies of the Kohn−Sham orbitals were allowed using the Gaussian smearing method and a width of 0.05 eV. The electronic energy was considered self-consistent when the energy change was smaller than 10^{-4} eV. A geometry optimization was considered convergent when the energy change was smaller than 0.05 eV $Å^{-1}$. In our structure, the U correction is used for Co and Mo atoms. The vacuum spacing in a direction perpendicular to the plane of the structure is 20 Å for the surfaces. The Brillouin zone integration is performed using 2×2×1 Monkhorst-Pack k-point sampling for a structure. Finally, the adsorption energies (E_{ads}) were calculated as $E_{ads}= E_{ad/sub}-E_{ad} -E_{sub}$, where $E_{ad/sub}$, E_{ad}, and E_{sub} are the total energies of the optimized adsorbate/substrate system, the adsorbate in the structure, and the clean substrate, respectively. The free energy was calculated using the equation:

$$G=E_{ads}+ZPE-TS$$

where G, E_{ads}, ZPE and TS are the free energy, total energy from DFT calculations, zero-point energy and entropic contributions, respectively.

Supplementary Figures

The as-prepared Co(OH)F precursor has a rodlike structure with a diameter of about 150 nm (Supplementary Fig. 1). Co(OH)F undergoes high-temperature calcination to remove hydrogen and fluorine, and the rodlike structure becomes a beaded-like structure (Supplementary Fig. 2a~c). The diameter of the material becomes c.a. 90 nm (inset Supplementary Fig. 2b). Furthermore, the defluorination and dehydrogenation of Co(OH)F during high-temperature calcination produces a lot of defects and oxygen vacancy (V_O).[S9-S11] The crystal interplanar spacing of 0.24 nm corresponds to the (111) plane of CoO (Supplementary Fig. 2d). The results of energy dispersive X-ray spectroscopy (EDS) mapping analysis (Supplementary Fig. 2e) further verified the typical beaded-like nanostructures, which clearly showed that Co and O are uniformly distributed throughout the beaded-like nanostructures.

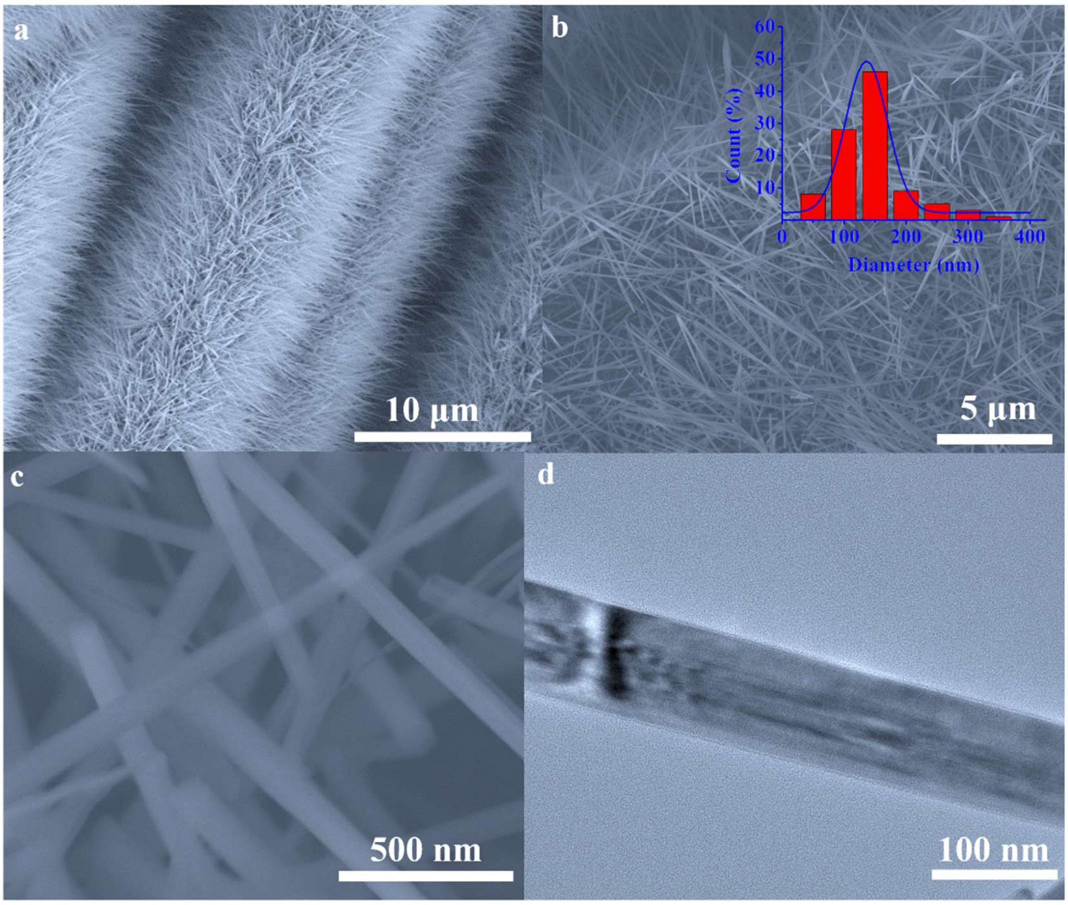

Supplementary Fig. 1. Morphology analysis of rodlike Co(OH)F. (a~c) SEM and (d) TEM images of Co(OH)F/CC.

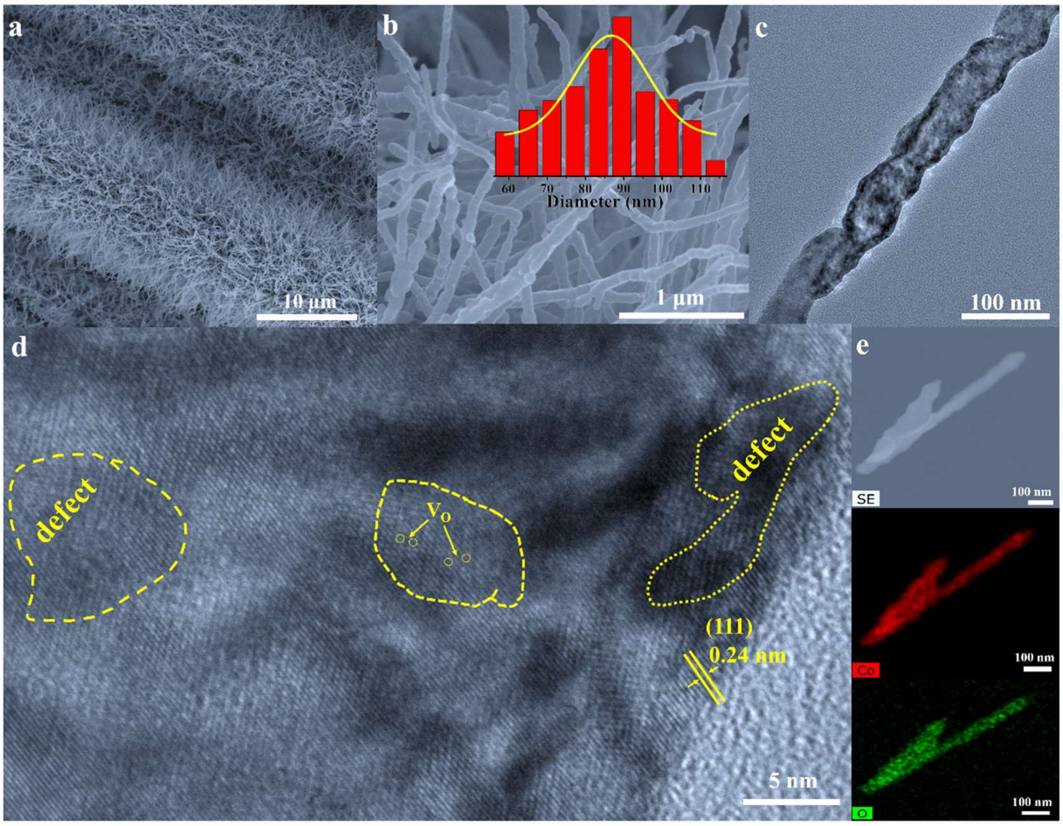

Supplementary Fig. 2. Morphology analysis of beaded-like CoO. (a, b) SEM, (c) TEM, (d) HRTEM and (e) EDS-Mapping images of CoO/CC. The illustration in Figure b is the diameter distribution of beaded CoO.

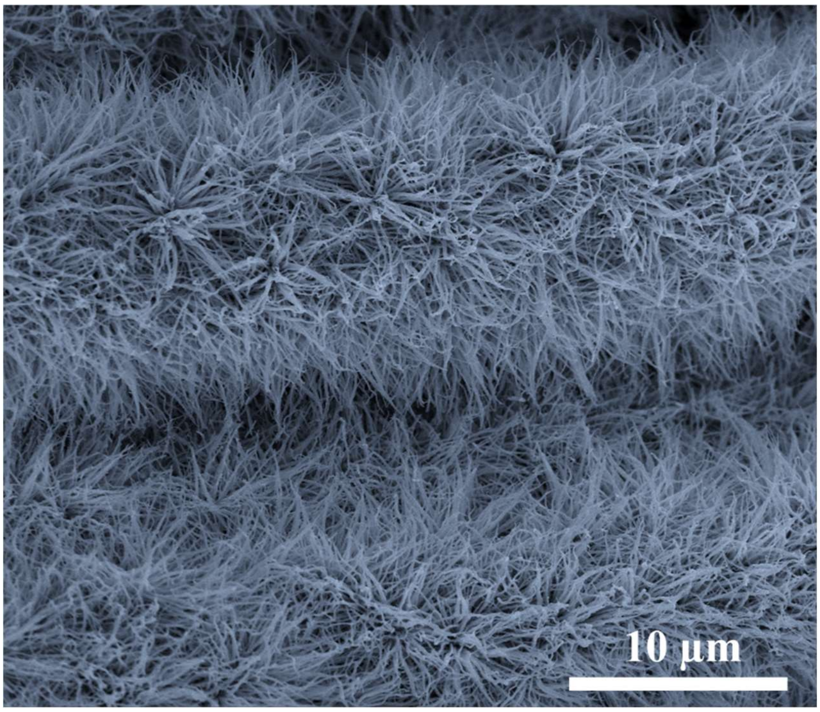

Supplementary Fig. 3. Morphology analysis of cowpea-like MoO_3@CoO/CC. SEM images of MoO_3@CoO/CC.

There are only corresponding elements for various target material in the full XPS spectrum (Supplementary Fig. 4), which is consistent with the results of DES-Mapping analysis.

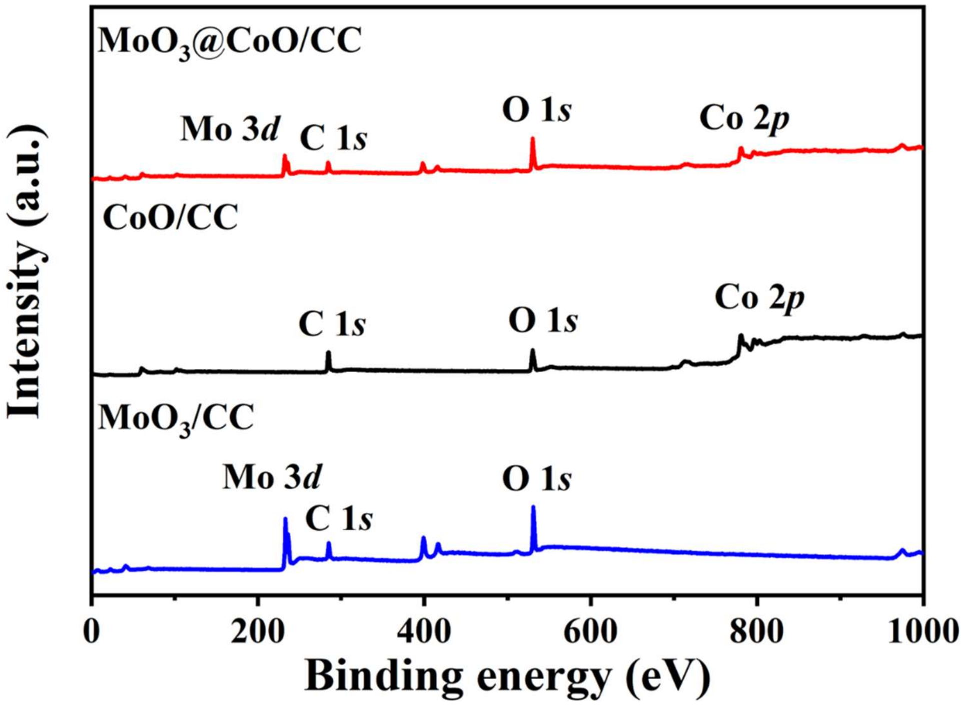

Supplementary Fig. 4. XPS analysis. XPS survey of the MoO_3@CoO/CC, CoO/CC, and MoO_3/CC catalysts.

In addition, with the increase of the number of deposition cycles (within 100~500 cycles), the catalytic activity of OER and the ability to shield chlorine is enhanced (Supplementary Fig.5). The OER exhibits maximum catalytic activity after deposition of MoO_3 for 500 cycles (thickness c.a. 5 nm). Subsequently, the catalytic activity of the catalyst decreases with increasing the number of deposition cycles (within 500~1000 cycles). This decrease can be attributed to the excessively thick shielding layer, which reduces the active area of the catalyst (The Supplementary Fig.6 supports this hypothesis).

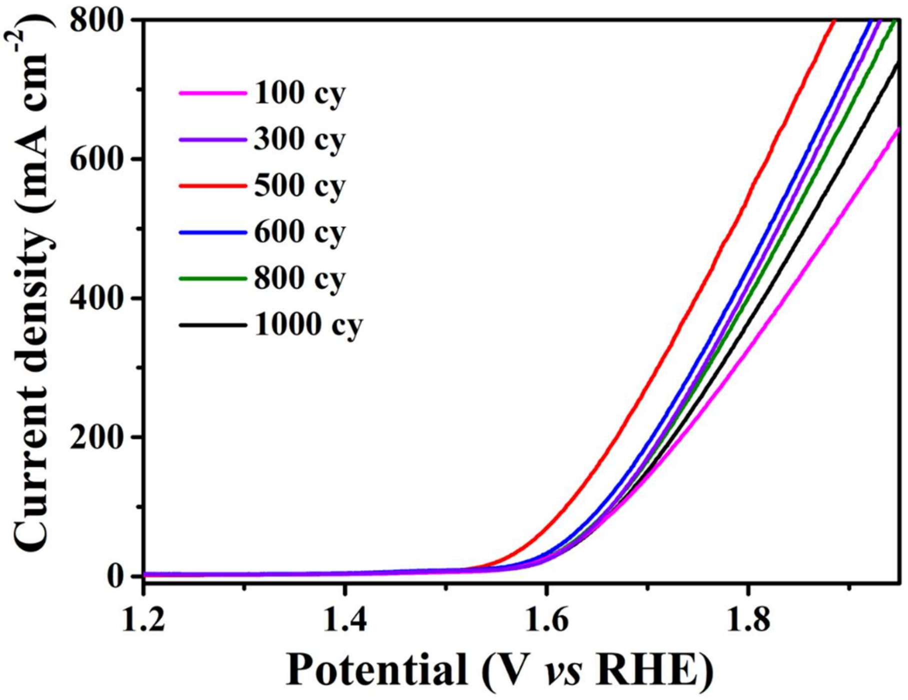

Supplementary Fig. 5. Polarization curve. The OER LSV curves of composite material MoO_3@CoO/CC bearing different ALD MoO_3 cycles.

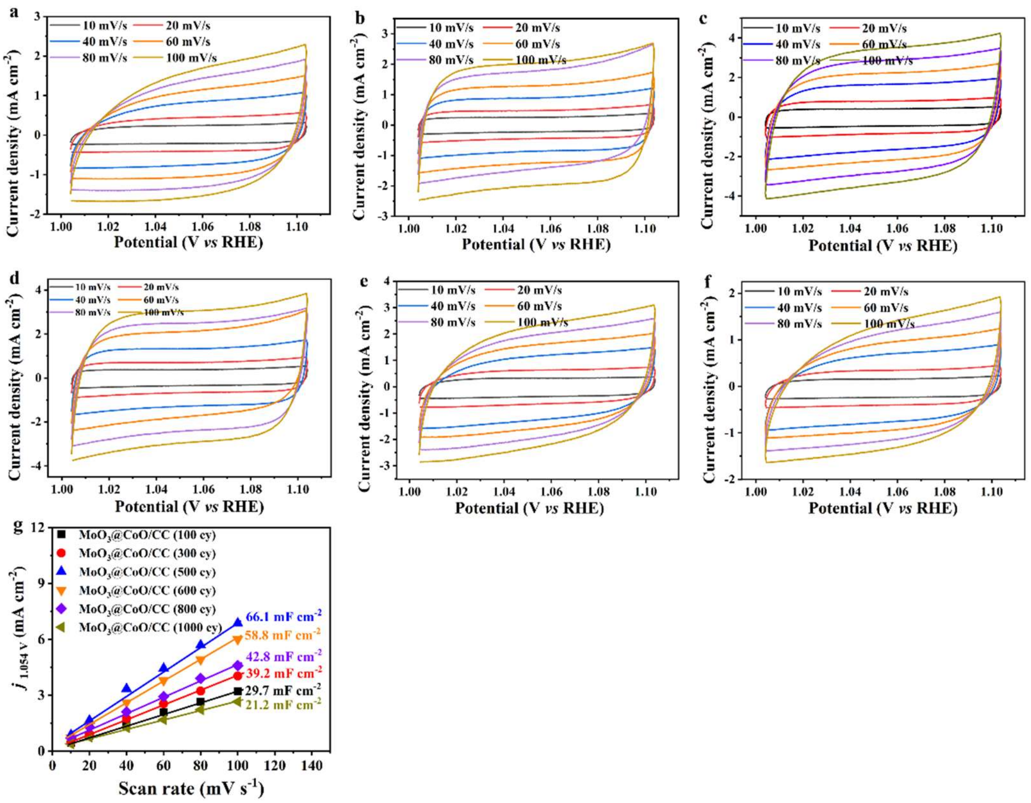

Supplementary Fig. 6. Electric double layer capacitance test. The cyclic voltammograms (CV) of (a) MoO_3@CoO/CC-100 cy, (b) MoO_3@CoO/CC-300 cy, (c) MoO_3@CoO/CC-500 cy, (d) MoO_3@CoO/CC-600 cy, (e) MoO_3@CoO/CC-800 cy and (f) MoO_3@CoO/CC-1000 cy performed in a non-Faradaic regime at different scan rates. (g) Plots of capacitive currents *vs* different scan rates with calculated double-layer capacitances C_{dl}.

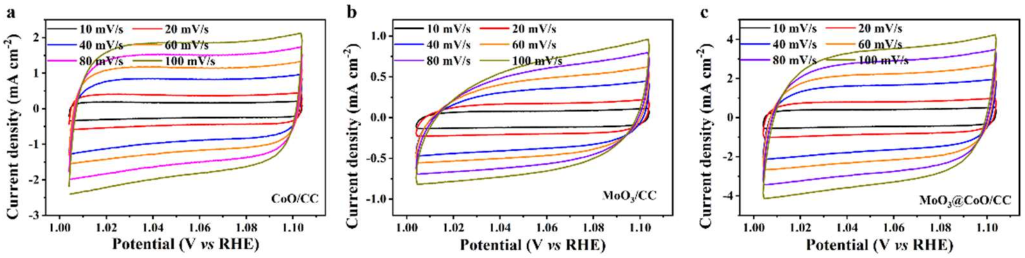

Supplementary Fig. 7. Cyclic voltammetric curve testing of various samples. The cyclic voltammograms (CV) of (a) CoO/CC, (b) MoO_3/CC and (c) MoO_3@CoO/CC performed in a non-Faradaic regime at different scan rates.

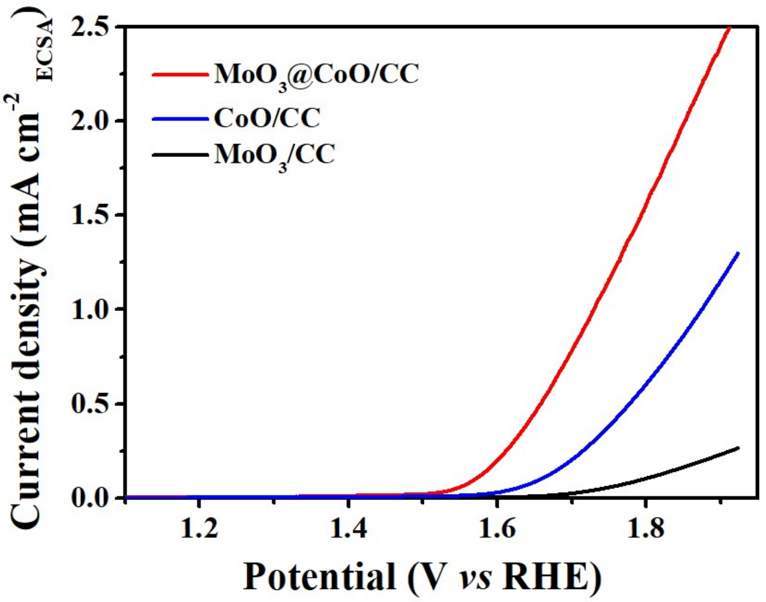

Supplementary Fig. 8. Electrochemical active area (ESCA) normalization. ECSA-normalized OER activity.

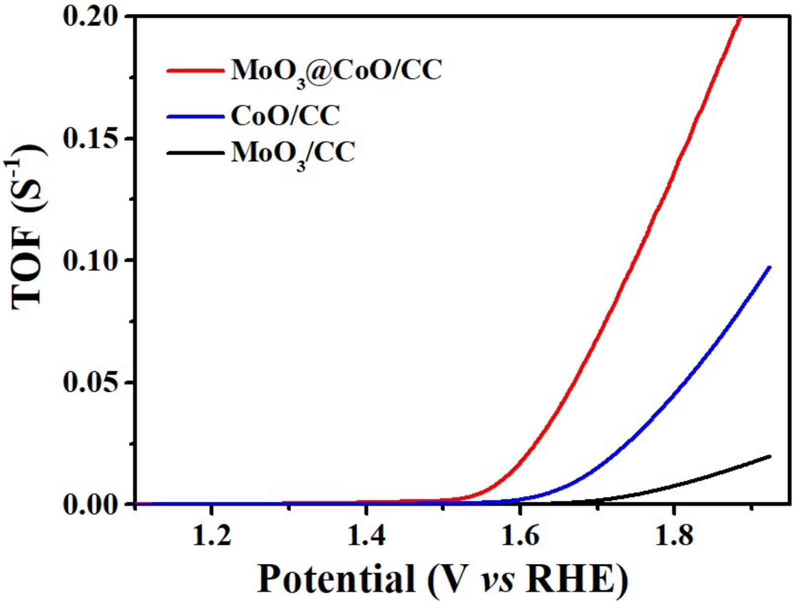

Supplementary Fig. 9. The turnover frequency calculation. Turnover frequency (TOF) plots of materials studied.

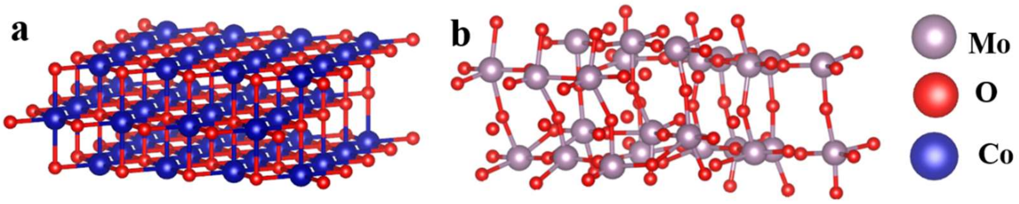

Supplementary Fig. 10. Density functional theory (DFT) calculation model. The CoO/CC, and MoO_3/CC optimized heterojunction structure.

As the ultra-thin MoO_3 layer is deposited on the surface of CoO, the adsorption energy of the catalyst to reactants/intermediates is effectively regulated, thereby the migration energy barrier of H_2O/OH at the MoO_3@CoO/CC interface lower.

After the MoO_3@CoO/CC adsorbs H_2O/OH, the charge at the interface is more concentrated than that at the CoO/CC interface (Supplementary Fig.11), indicating the high activity of MoO_3@CoO/CC catalyst for H_2O/OH. After MoO_3@CoO/CC adsorbs Cl^-, the charge at the interface are dispersed, which indicates that MoO_3@CoO/CC catalyst exhibits lower activity for Cl^-.

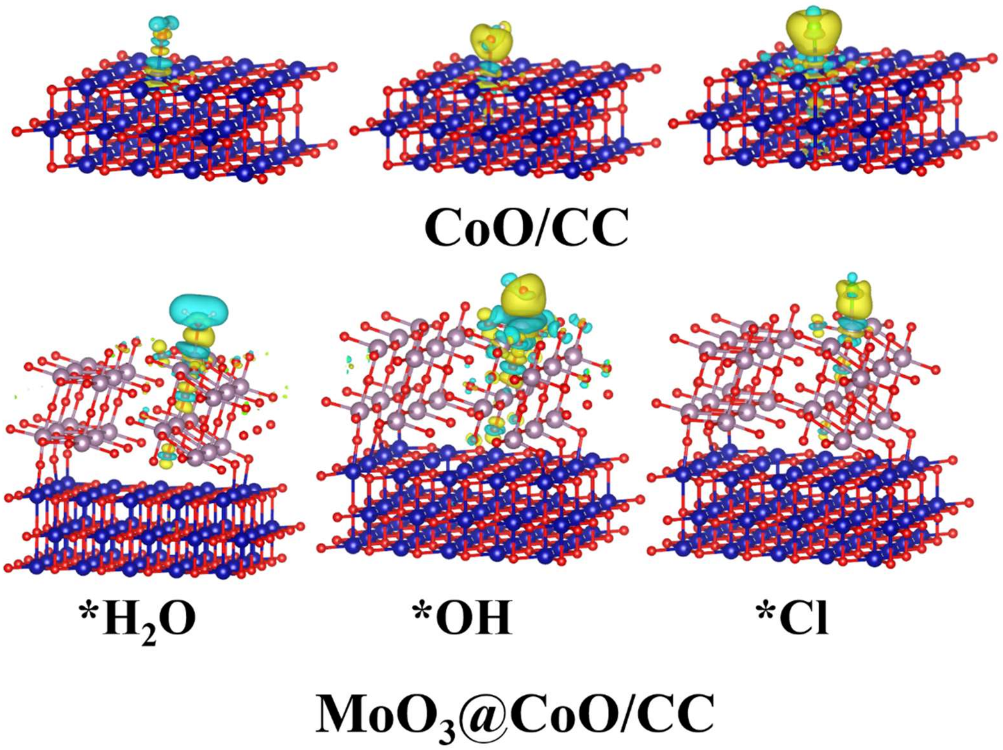

Supplementary Fig. 11. The differential charge density after adsorption of various small molecules. The differential charge density of CoO/CC and MoO_3@CoO/CC structure adsorbing *H_2O, *OH and *Cl. The equivalent surface value is 0.002 eV/Å^3.

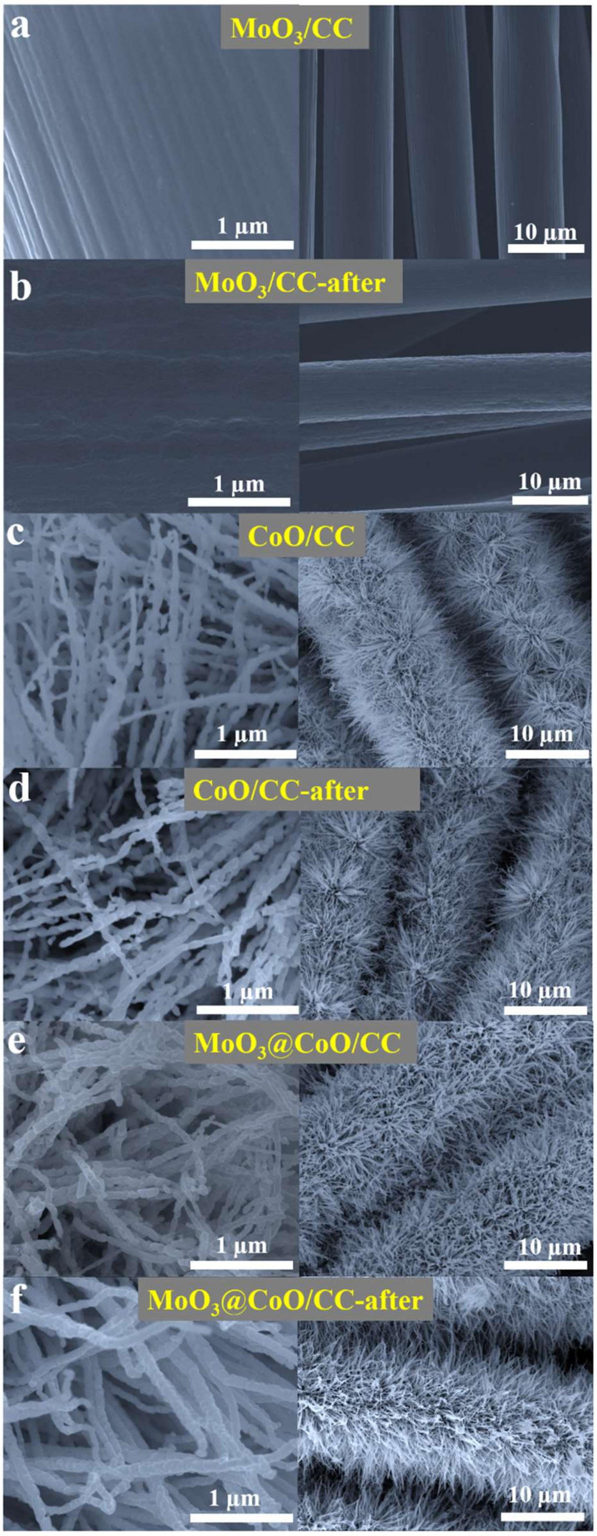

Supplementary Fig. 12. Morphology analysis of various samples after OER in seawater. SEM images of (a) MoO_3CC, (b) MoO_3/CC-after, (c) CoO/CC, (d) CoO/CC-after, (e) MoO_3@CoO/CC and (f) MoO_3@CoO/CC-after.

The electron energy loss spectrum (EELS) of the MoO_3@CoO/CC-after catalysts as shown in Supplementary Fig. 13. As shown in Supplementary Fig. 13a, the green and dark yellow wireframes are the surface scan and error margin, respectively. As shown in Supplementary Fig. 13b, the outermost layer, the interfacial and intermediate layer of the MoO_3@CoO-after sample was selected, correspond to the MoO_3, CoMo-LDH and CoO, respectively. In Supplementary Fig. 13c, the peak at 535.5 eV detected in the MoO_3 layer corresponds to the Mo-O bond in the amorphous structure.[S12] The shoulder peak at 531 eV is attributed to the hybridisation of O 2p with Co 3d and Mo 3d orbitals.[S12] It is worth noting that the hybridization intensities between O 2p and M 3d orbitals is weak, which is mainly due to the influence of -O-O- structure.[S13,S14] The Co-L3 edge of the interfacial layer is blue-shifted by about 0.6 eV compared to the intermediate-phase CoO, indicating a higher Co valence.[S13] Similarly, the Mo edge (Supplementary Fig. 13d) of the interface layer is blue shifted by about 2.0 eV compared to the MoO_3 layer, indicating that the MoO_3 is partially reconstructed.[S14,S15] The above results further verify the existence of CoMo-LDH.

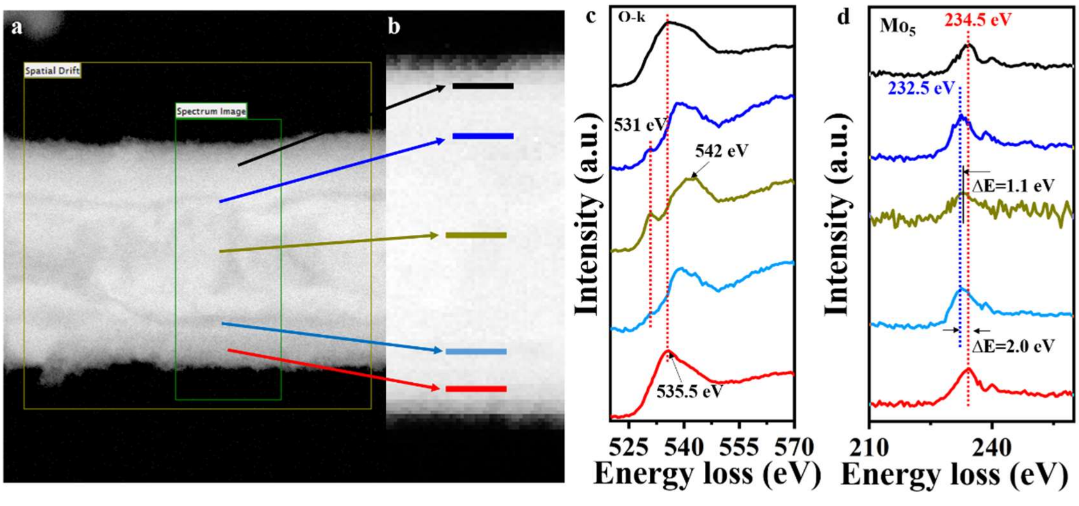

Supplementary Fig. 13. Electron energy loss spectrum analysis of MoO_3@CoO/CC-aft catalyst. (a) HAADF image of MoO_3@CoO after continuous oxygen evolution in seawater for 50 h. (b) EELS Spectrum Image (low-loss). EELS spectra of (c) O-K, and (d) Mo M-edge of MoO_3@CoO/CC-after.

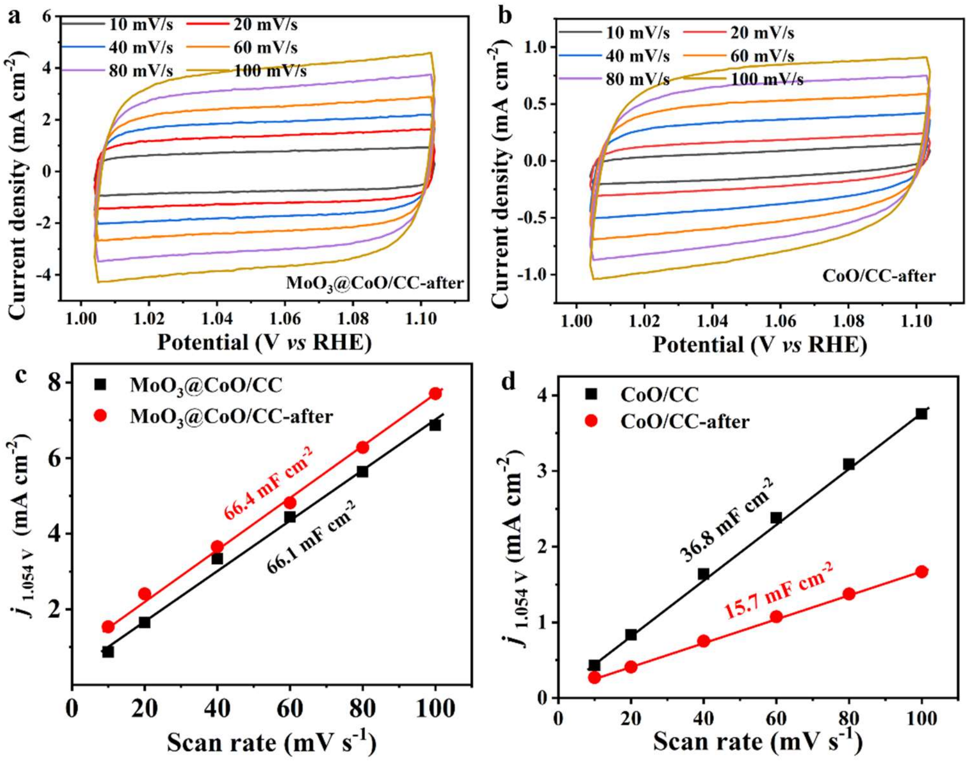

Supplementary Fig. 14. Measurement of electric double layer capacitance after seawater oxidation. The cyclic voltammograms (CV) of (a) MoO_3@CoO/CC-after and (b) CoO/CC-after performed in a non-Faradaic regime at different scan rates. (c) The C_{dl} comparison plots of MoO_3@CoO/CC catalyst before and after the reaction. (d) The C_{dl} comparison plots of CoO/CC catalyst before and after the reaction.

In the Co 2*p* XPS spectrum, two new peaks of 780.6 and 794.9 eV were observed after the OER reaction (Supplementary Fig. 15a). Simultaneously, the binding energies of 780.2 and 796.0 eV for Co^{2+} shifted significantly negatively after OER reaction. This is mainly due to the chemical state change of cobalt caused by the gradual oxidation of Co^{2+} to Co^{4+} in the OER process.[S16, S17] In addition, the Mo-O bond also shifts negatively after OER reaction, which may be caused by the bonding between Cl^- and Mo in seawater (Supplementary Fig. 15b).

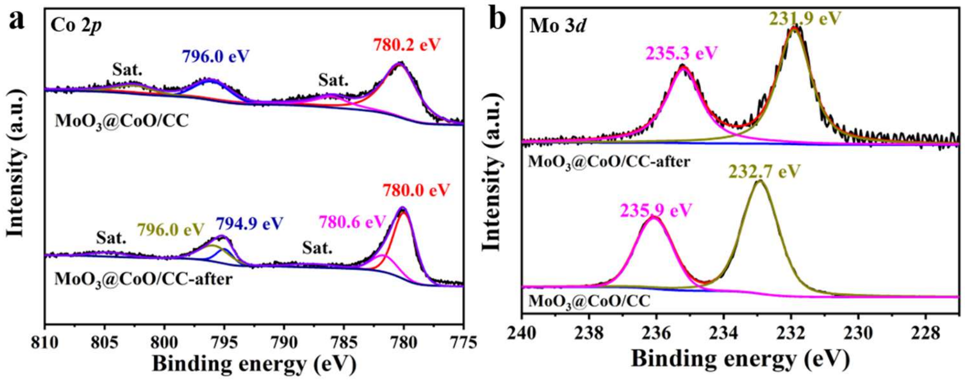

Supplementary Fig. 15. Structural analysis of various samples after OER in seawater. High-resolution XPS spectra of (a) Co 2p and (b) Mo3d before and after the OER of MoO_3@CoO/CC catalyst.

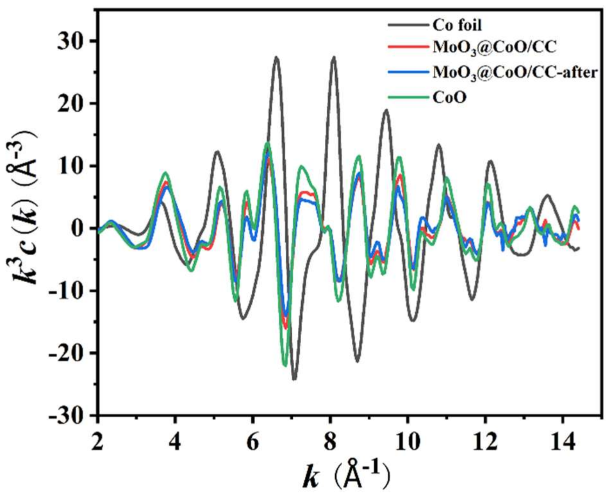

Supplementary Fig. 16. X-ray absorption near edge structure (XANES) analysis. k space fitting curve of CoO, MoO_3@CoO/CC, MoO_3@CoO/CC-after and CoO foil.

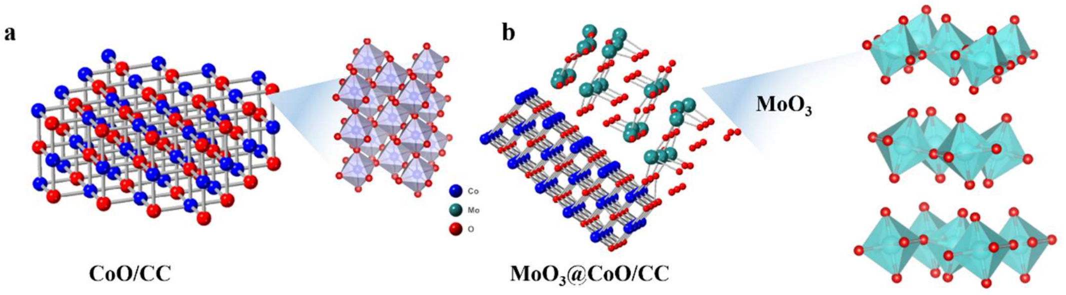

Supplementary Fig. 17. K-space fitting structure model. The (a) CoO/CC and (b) MoO_3@CoO/CC optimized structure.

As can be seen from Supplementary Fig. 18, the Co-O-Co bond exhibits the strongest signal clearly detected at $k \approx 6.22$ $Å^{-1}$ for the CoO sample. The introduction of MoO_3 on CoO surface will obviously change the position of the strongest signal, which further indicates that the new Co-O-Mo bond change the coordination of Co atoms. After OER, the strongest signal of MoO_3@CoO/CC-after obviously red-shifts due to the oxidation of partial Co at the interface to high valence state. In addition, specific structural parameters are obtained by quantitative fitting EXAFS of various samples (Supplementary Fig. 19). Compared with CoO, the Co-O signal of MoO_3@CoO/CC splits into two peaks, which indicates that a new Co-O-Mo bond is formed in MoO_3@CoO/CC. Compared with MoO_3@CoO/CC, the peak position of Co-O in MoO_3@CoO/CC-after changed from 1.38, 1.69 Å to 1.41 and 1.78 Å, respectively, indicating that a new phase was formed on the Co surface in MoO_3@CoO/CC-after.

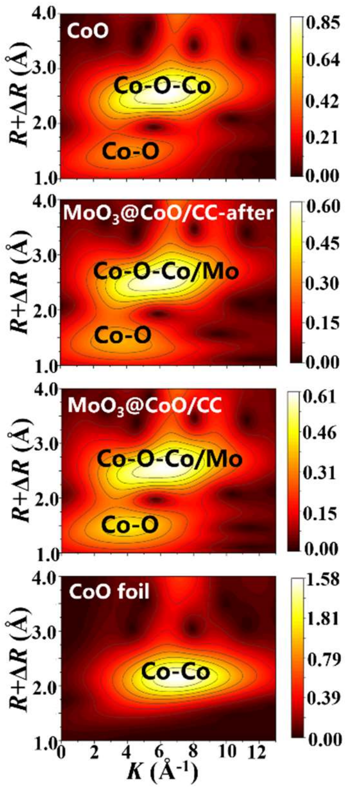

Supplementary Fig. 18. Wavelet transform (WT)-EXAFS analysis of Co K-edge. WT-EXAFS images of various Co-based samples.

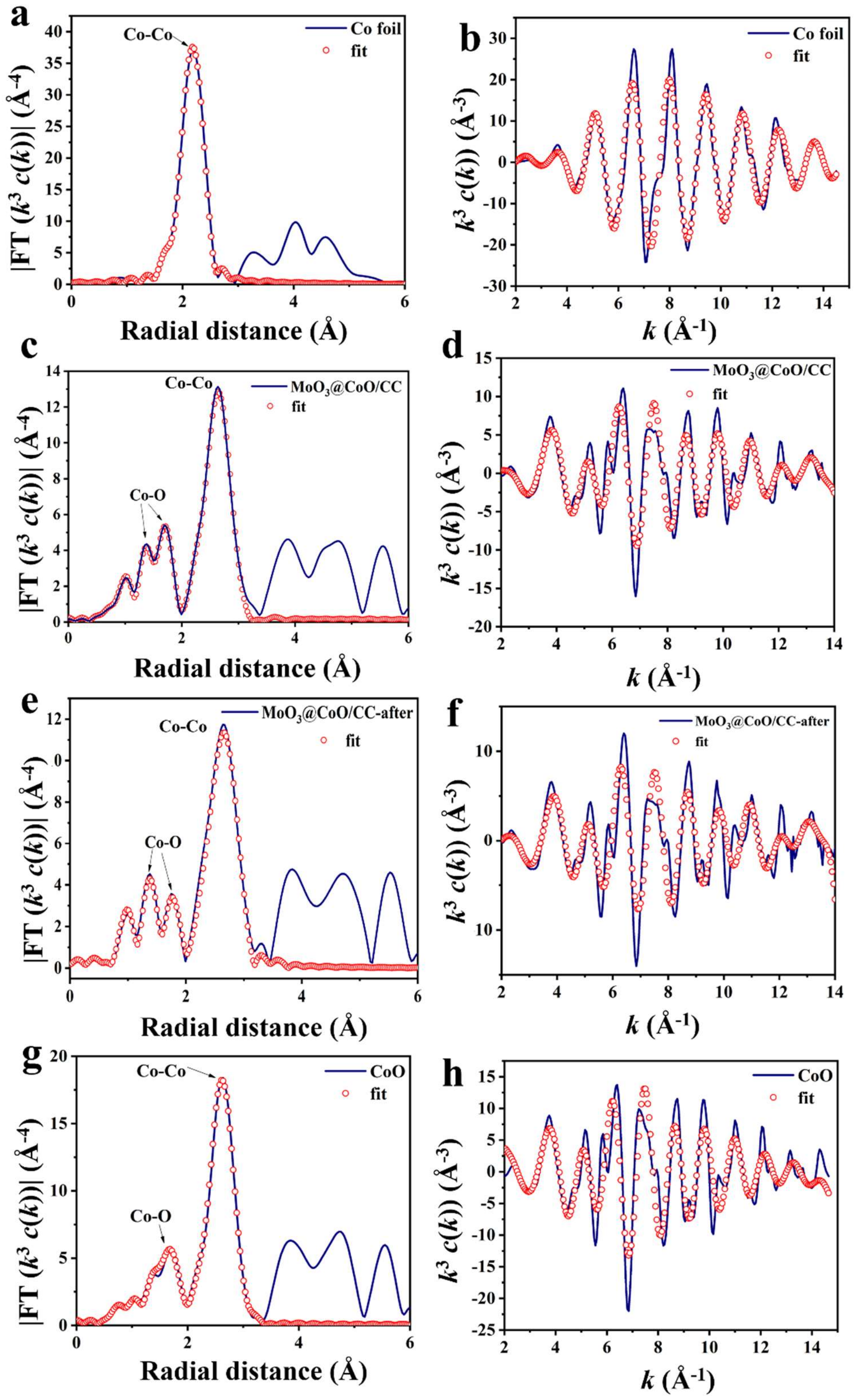

Supplementary Fig. 19. Co-k edge k space fitting curves. R space fitting curves of (a, b) Co foil, (c, d) MoO_3@CoO/CC, (e, f) MoO_3@CoO/CC-after and (g, h) CoO at Co K-edge.

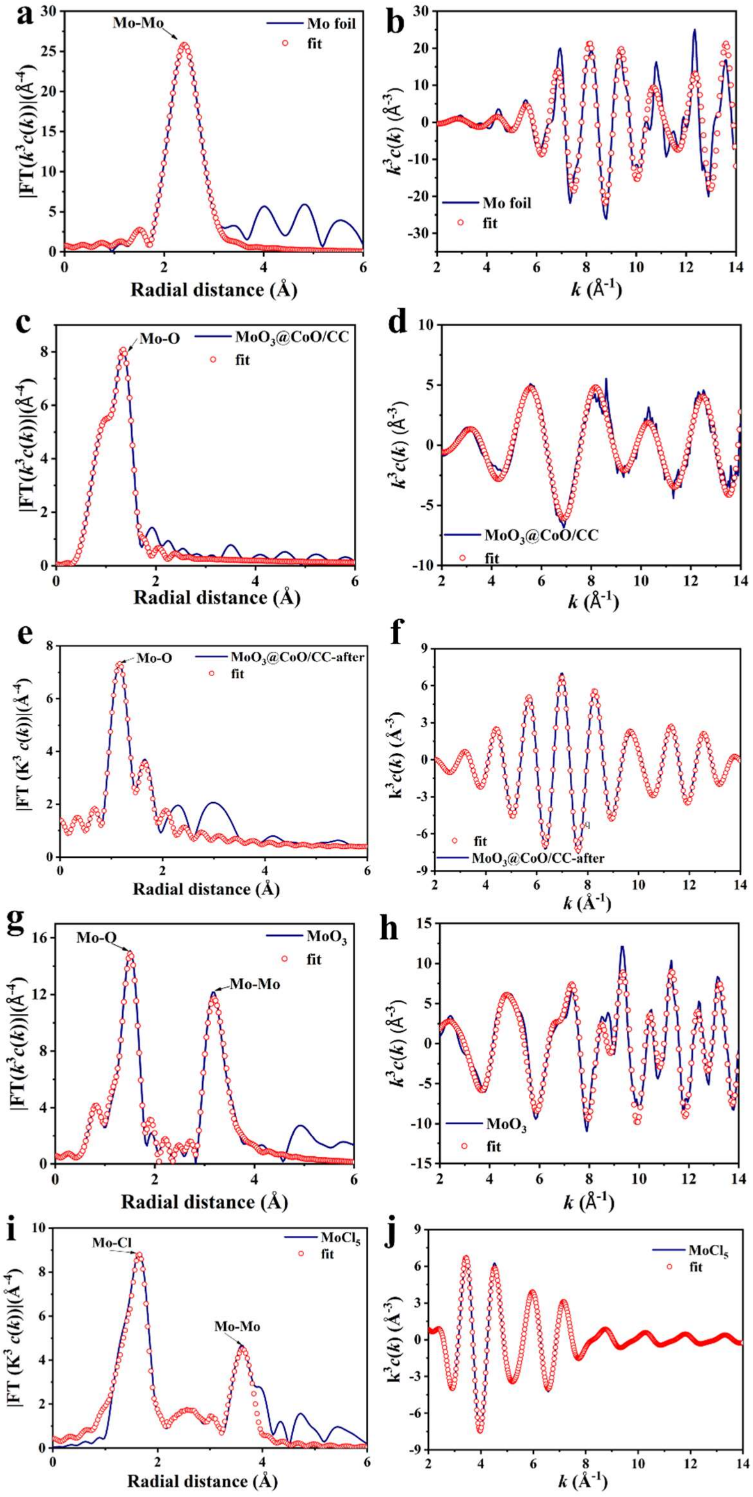

Supplementary Fig. 20. Mo-k edge k space fitting curves. R space fitting curves of (a, b) Mo foil, (c, d) MoO_3@CoO/CC, (e, f) MoO_3@CoO/CC-after, (g, h) MoO_3 and (i, j) $MoCl_5$ at Mo K-edge.

Notably, due to the bond signals of Mo-O-Co and Mo-O are close, they are collectively referred to as Mo-O-Co/Mo-O. Supplementary Fig. 21 shows a circular Mo-O bond signal at 4.67 Å^{-1}. Due to the existence of Mo-O-Co bond in MoO_3@CoO/CC, the bond length of the coordination atom becomes large, resulting in the strongest signal appearing at 5.67 Å^{-1}. The strongest signal region of MoO_3@CoO/CC-after became irregular compared to MoO_3@CoO/CC, which is mainly due to the formation of new Mo-O-O and Mo-Cl bonds in MoO_3@CoO/CC-after during the OER reaction.

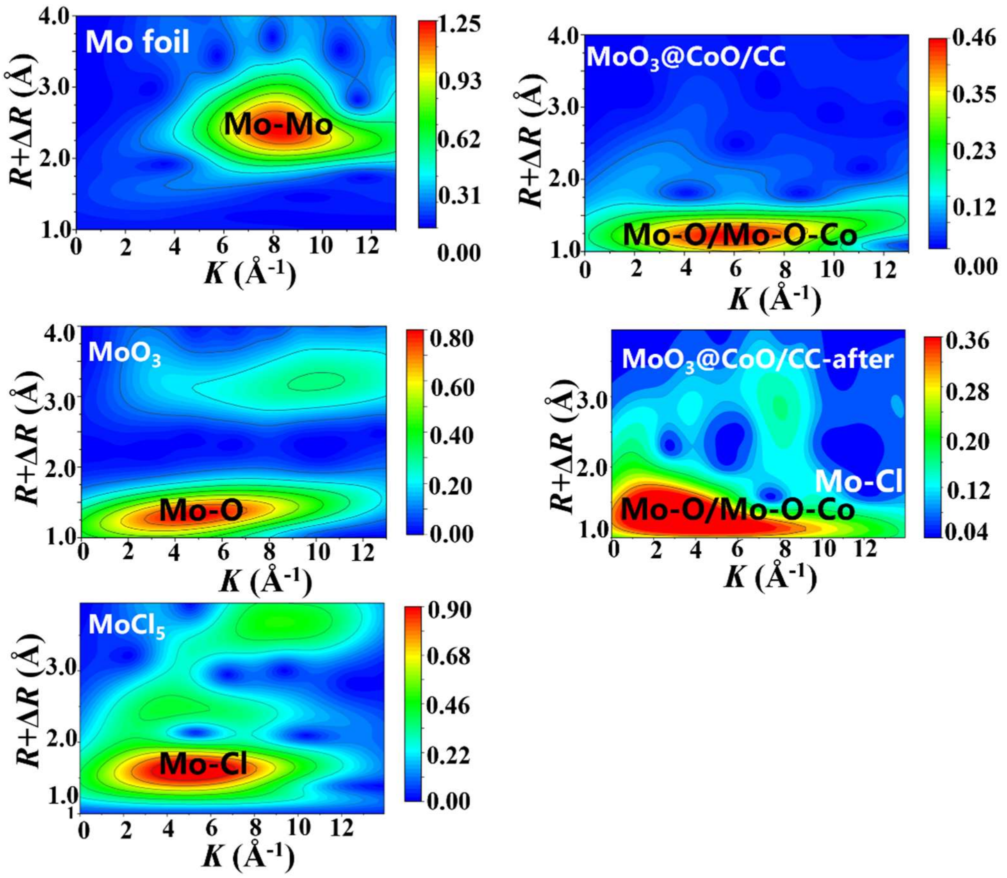

Supplementary Fig. 21. Wavelet transform (WT)-EXAFS analysis of Mo K-edge. WT-EXAFS images of various Mo-based samples.

The intermediates of H_2O in OER process are M-OH, M-O and MOOH,[S18] respectively. Among them, oxygen can be produced by the direct diffusion and reaction of two M–O intermediates, or by the decomposition of the adsorbed M–OOH intermediate in electrolyte. Interestingly, M-OOH intermediates are usually active substances in OER process. When multiple metals with matching crystal types coexist, multi-metal layered double hydroxides (MLDH) are formed by phase fusion.[S19] For this reason, the OER of MoO_3@CoO/CC catalyst in seawater may self-reconstruct to form CoMo LDH phase. This CoMo LDH layered compound is composed of MO_6 (M=Co, Mo) octahedral structure. In the absence of MoO_3 layer, both chlorine evolution reaction (CER) and OER are present in the CoO in the electrolytic seawater. The initial catalysis of MoO_3@CoO/CC is the adsorbate evolution mechanism (AEM).

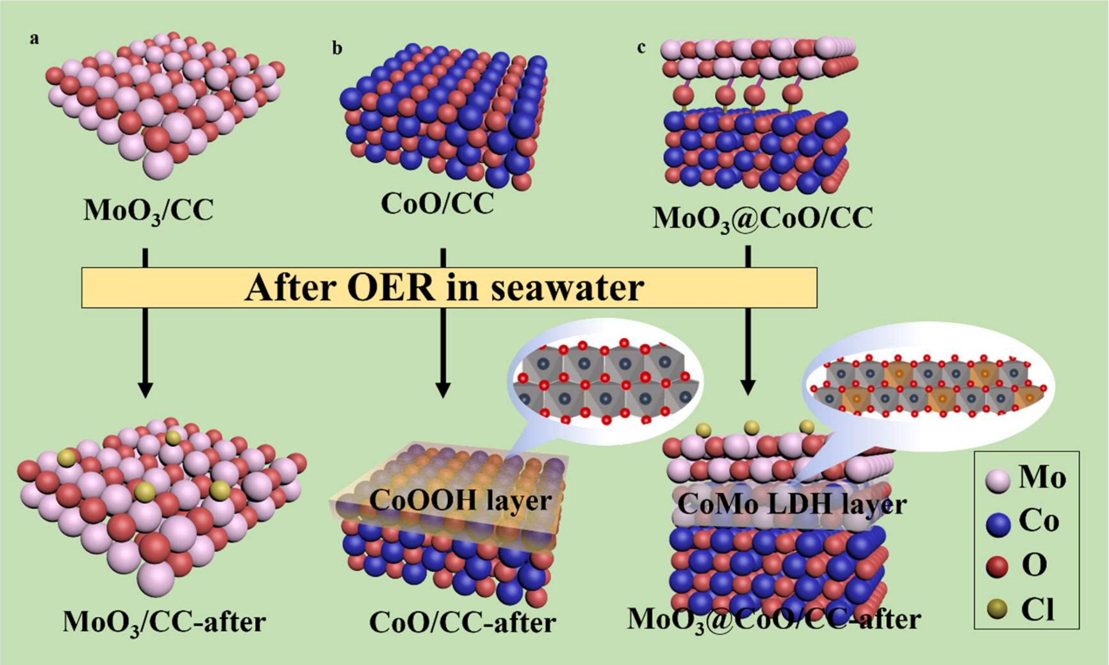

Supplementary Fig. 22. Schematic diagram of structural transformation of various samples. Schematic images of (a) MoO_3CC and MoO_3/CC-after, (b) CoO/CC and CoO/CC-after, (c) MoO_3@CoO/CC and MoO_3@CoO/CC-after.

The effect of ultra-thin MoO_3 layer on the mass transfer process of cowpea-like MoO_3@CoO/CC was investigated by ion diffusion experiment (Supplementary Fig. 23). Obviously, the MoO_3@CoO/CC catalyst much higher $I_P/v^{0.5}$ than CoO/CC catalyst (Fig. 6a), which strongly proves that the ultra-thin MoO_3 layer significantly promotes the mass transfer process.

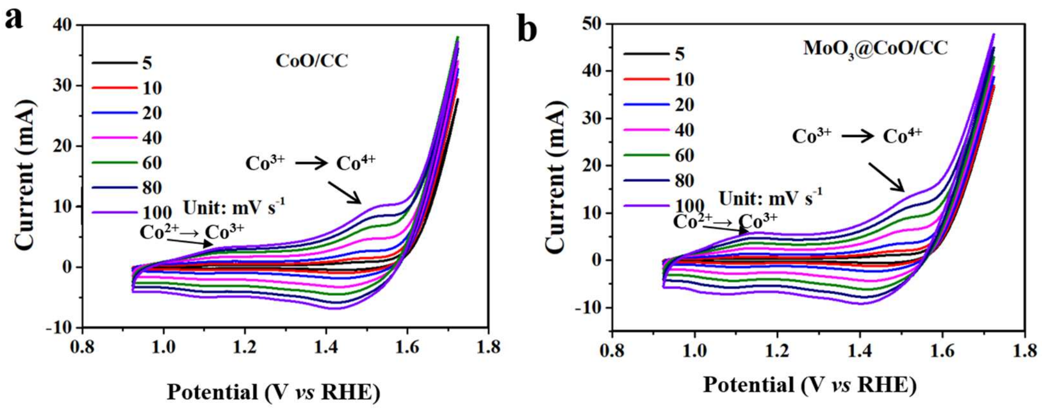

Supplementary Fig. 23. Diffusion dynamics analysis. (a) CV curves of CoO/CC. (b) CV curves of MoO_3@CoO/CC.

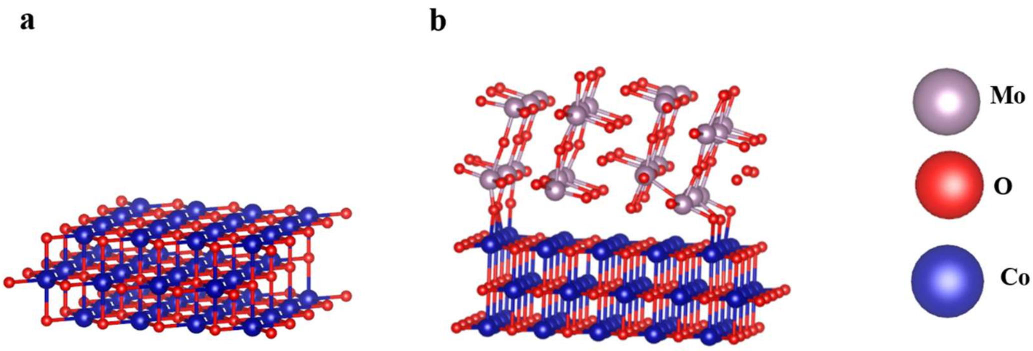

Supplementary Fig. 24. DFT calculation model. The (a) CoO/CC and MoO_3@CoO/CC optimized heterojunction structure.

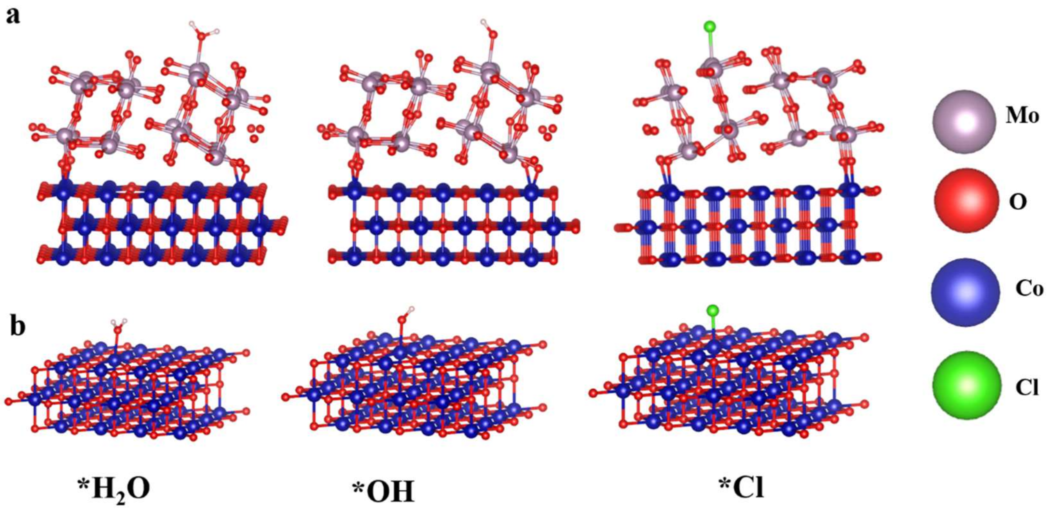

Supplementary Fig. 25. DFT calculation adsorption model. The adsorption structure of (a) MoO_3@CoO/CC and (b) CoO/CC for *H_2O, *OH and *Cl, respectively.

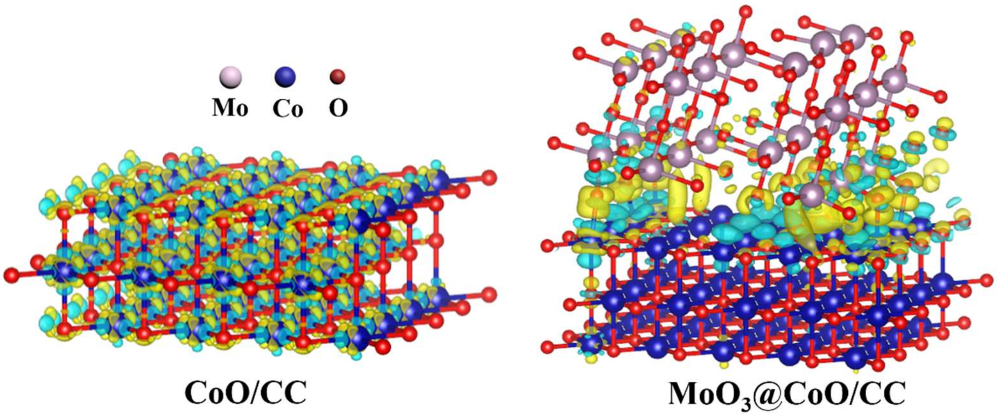

Supplementary Fig. 26. The differential charge density. The differential charge density of CoO/CC and MoO_3@CoO/CC structure. The equivalent surface value is 0.003 eV/$Å^3$.

Generally, AEM and LOM pathways involve five basic steps such as four electrochemical steps and one O_2 desorption step. Due to the reconstructed CoMo LDH is confined between CoO and MoO_3, and the existence of Co-O-Mo bond leads to the decrease of the density of the 2p orbital electron cloud of O. Based on this, the intermediate produced in step A2 in AEM mechanism can easy to form the epoxy compound intermediate in LOM mechanism (Supplementary Fig.27). Notably, the formation of epoxy compounds is the decisive step of LOM mechanism. Subsequently, through the L1 step, -O-(OH)Co-O2-MoO3 is formed, and O is further evolved by reaction (Step L2), thus improving the reaction kinetics.

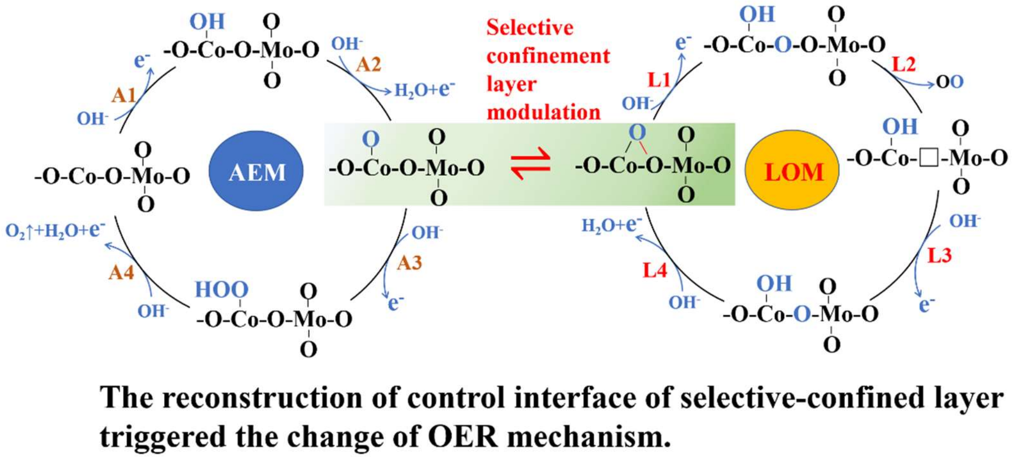

Supplementary Fig. 27. Schematic diagram of the catalytic mechanism. Scheme of electrolysis mechanism of seawater with MoO_3@CoO/CC catalyst.

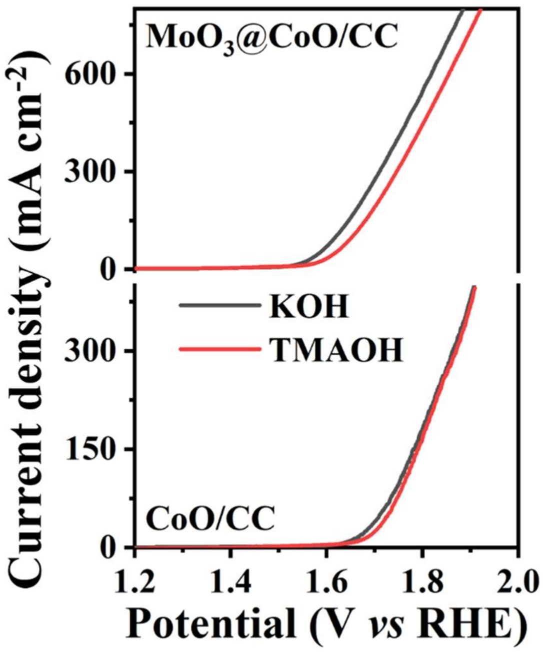

Supplementary Fig. 28. The influence of TMAOH for various catalysts. (a) LSV curves of MoO_3@CoO/CC and CoO/CC in 1.0 M KOH and 1.0 M TMAOH.

Mass spectra of the gas products showed that no signal for Cl_2 appeared at m/z = 71. We could also rule out ClO^- generation at the anode since it would react with Cl^- in solution via, Cl^- (aq) + ClO^- (aq) + H_2O (l) → Cl_2 (g) + $2OH^-$ (aq). The lack of chloride oxidation was consistent with the high selectivity for OER evidenced by Faradaic efficiency measurements by gas chromatography (Supplementary Fig. 29a). We defined a relative Faradaic Efficiency (RFE) as the ratio of oxygen generation in various electrolyte to a 1 M KOH electrolyte (Supplementary Fig. 29b), as OER with SFCNF/$Co_{1-x}S$@CoN catalysts are known to have a Faradaic efficiency of nearly 100% in 1 M KOH electrolytes.[S19] Actually, the MoO_3@CoO/CC showed the same OER-FE in various electrolytes, confirming high selectivity for OER in the presence of NaCl.

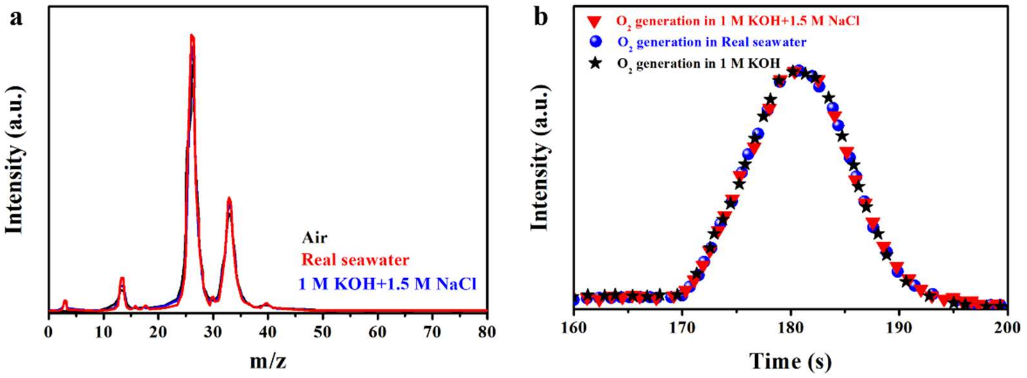

Supplementary Fig. 29. Analysis of chlorine evolution and oxygen production efficiency of catalysts in various electrolytes. The mass spectra of the products were run on sample MoO_3@CoO/CC at a current density of 400 mA cm^{-2} in various electrolytes. (b) Gas chromatographic O_2 signal collected from the electrolyzer running at 400 mA cm^{-2} in 1 M KOH, real seawater and 1 M KOH + 1.5 M NaCl.

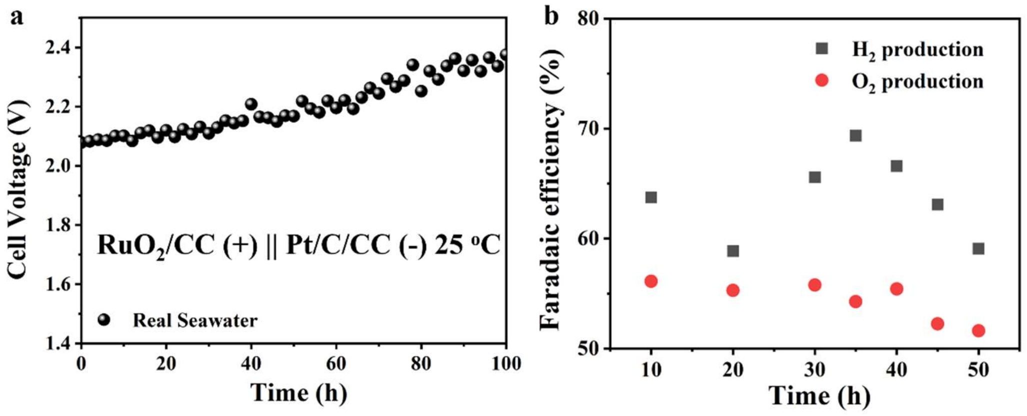

Supplementary Fig. 30. Performance of two-electrode system. (a) Continuous electrolytic stability of RuO_2/CC (+) || Pt/C/CC (-) 25 °C. (b) Faraday efficiency (FE) of producing H_2 and O_2, respectively.

It is worth noting that in a flow cell seawater enters from the anode side and passes through the exchange film to the cathode side (see Supplementary Fig. 31). Due to the anode is made of MoO_3@CoO/CC material, it has high selectivity for Cl^- and can effectively inhibit CER, thus realizing direct seawater oxidation. In addition, because MoO_3 has an octahedral structure of MoO_6, oxygen atoms are placed at the top of the octahedron, which have a good adsorption capacity for seawater cations, thus avoiding the cations from reaching the cathode and improving the catalytic stability of the Pt/C/CC electrode. As shown in Fig. S30, the RuO_2/CC (+) || Pt/C/CC electrolytic cell shows poor stability and Faraday efficiency, indicating that cations and anions in seawater have great influence on the catalyst. The above analysis results further show that MoO_3@CoO/CC catalyst exhibits good direct seawater oxidation ability, and can adsorb cations in seawater in the flow electrolytic cell, thus protecting the cathode.

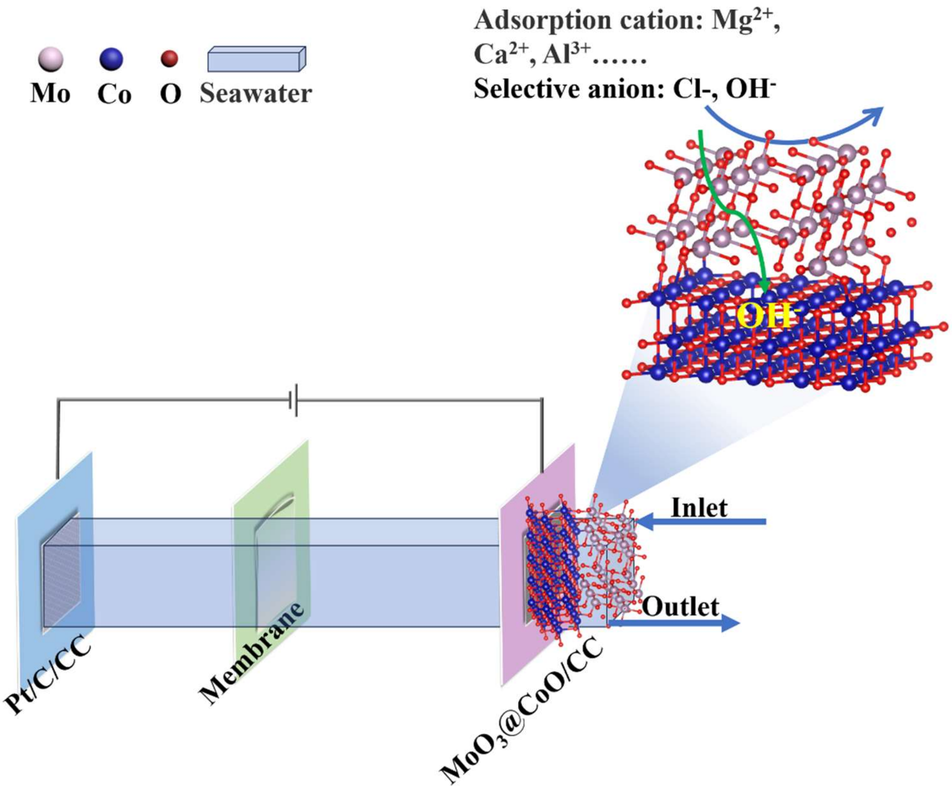

Supplementary Fig. 31. Characteristics analysis of flow electrolytic cell. Schematic diagram of seawater in and out of the flow electrolytic cell and the effect of MoO_3@CoO/CC on seawater.

Supplementary Table 1. AC impedance fitting. Impedence parameters of various materials calculated from equivalent circuit model.

Sample	CPE (Ω)	Rct (Ω)	Rs (Ω)
**MoO_3@CoO/CC**	0.82	2.0	1.7
**CoO/CC**	0.90	2.4	1.9
**MoO_3/CC**	0.95	6.1	1.8

Supplementary Table 2. Performance comparison. Performance comparison of non-noble metal OER electrocatalysts in seawater.

Material	Tafel slope (mV dec^{-1})	Long-term stability@η_{100}	Ref.
**MoO_3@CoO/CC**	**55**	**100 h**	**This work**
Fe-$Ni(OH)_2/Ni_3S_2$@NF	46	27 h	S20
Co_3O_4-MnO_2	41	1 h	S21
FNE300	45	50 h	S22
$Co_2(OH)_3Cl$	58.5	60000 s@η_{130}	S23
$Ni(OH)_2$-TCNQ/GP	75	80 h	S24
N-CDs/NiFe-LDH/NF	43.4	50@η_{15}	S25
NiFeCo-LDH	46	80 h	S26
Ni_2Fe-LDH/$FeNi_2S_4$/NF	55.9	20 h@η_{50}	S27
$Ir_1/Ni_{1.6}Mn_{1.4}O_4$	75	60	S28
0.5Fe-$NiCo_2O_4$@CC	76.1	20@$\eta_{21.7}$	S29
Ni_3S_2/Fe-NiP_x/NF	61.3	25 h	S30
Co-N, P-HCS	121.5	100 h	S31
$Ni_xB/B_4C/B$-C_{PR}/NF-B5P1	41	24 h	S32
Ni_2P-Fe_2P/NF	58	24 h	S33
Co-Se1	40.4	12 h@η_{10}	S34

Supplementary Table 3. Structural parameters extracted from the EXAFS fitting. EXAFS fitting parameters at the **M** *K*-edge (M=Co, Mo) for various samples.

Sample	Shell	*CN*[a]	*R*(Å)[b]	σ^2(Å^2)[c]	ΔE_0(eV)[d]	*R* factor
Co *K*-edge (S_0^2=0.753)						
Co foil	Co-Co	12*	2.494±0.003	0.0061±0.0001	6.7±0.3	0.0029
CoO	Co-O	6.0±0.4	2.077±0.022	0.0102±0.0018	-2.1±0.9	0.0063
	Co-Co	11.8±0.5	3.016±0.005	0.0081±0.0008		
MoO_3@ CoO/CC	Co-O1	0.8±0.5	1.985±0.013	0.0082±0.0024	1.5±3.6	0.0081
	Co-O2	4.7±0.3	2.105±0.026			
	Co-Co	10.1±0.8	3.010±0.017	0.0094±0.0005	1.2±2.1	
	Co-O-Mo	1.2±0.3	3.198±0.024			
MoO_3@ CoO/CC-after	Co-O1	2.4±0.3	1.906±0.017	0.0052±0.0045	2.1±6.1	0.0137
	Co-O2	3.4±0.1	2.134±0.016			
	Co-Co	9.7±0.5	3.003±0.019	0.0105±0.0021	1.6±6.9	
	Co-O-Mo	0.8±0.3	3.213±0.016			
Mo *K*-edge (S_0^2=0.803)						
Mo foil	Mo-Mo	8*	2.721±0.005	0.0031±0.0008	-8.4±0.7	0.0005
	Mo-Mo	6*	3.124±0.003	0.0024±0.0008		
MoO_3	Mo-O	6.0±0.5	1.956±0.005	0.0034±0.0005	-2.4±1.0	0.0078
	Mo-Mo	5.0±0.7	3.651±0.004	0.0032±0.0004		
MoO_3@ CoO/CC	Mo-O	4.8±0.5	1.846±0.011	0.0106±0.0046	-3.4±6.8	0.0086
	Mo-Mo	5.0±0.2	3.147±0.005	0.0023±0.0006		
	Mo-O-Co	2.3±0.3	1.78±0.011	2.6±0.6	-6.7±0.6	
MoO_3@ CoO/CC-after	Mo-O	4.5±0.2	2.03±0.005	0.0031±0.0014	2.9±1.4	0.0043
	Mo-Mo	4.2±0.2	2.52±0.002	0.0056±0.0003		
	Mo-O-Co	2.4±0.1	1.04±0.08	3.17±0.046	-3.1±0.6	
	Mo-Cl	2.8±0.1	1.76±0.09	2.25±0.038		
$MoCl_5$	Mo-Mo	5.2±0.8	2.12±0.008	0.051±0.0007	-5.2±1.5	0.0025
	Mo-Cl	5.1±0.2	2.14±0.05	4.14±0.06		

[a]*CN*, coordination number; [b]*R*, the distance to the neighboring atom; [c]σ^2, the Mean Square Relative Displacement (MSRD); [d]ΔE_0, inner potential correction; *R* factor indicates the goodness of the fit. S_0^2 was fixed to 0.753 and 0.803, according to the experimental EXAFS fit of Co foil and Mo foil by fixing *CN* as the known crystallographic value. * This value was fixed during EXAFS fitting, based on the known structure of Co and Mo. Fitting range: 3.0 ≤ *k* (/Å) ≤ 14.0 and 1.0 ≤ *R* (Å) ≤ 3.0 (Co foil); 2.0 ≤ *k* (/Å) ≤ 13.6 and 1.0 ≤ *R* (Å) ≤ 3.5 (MoO_3@CoO/CC); 2.0 ≤ *k* (/Å) ≤ 13.5 and 1.0

$\leq R$ (Å) ≤ 3.5 (MoO_3@CoO/CC-after); $3.0 \leq k$ (/Å) ≤ 12.0 and $1.0 \leq R$ (Å) ≤ 3.0 (Mo foil); $2.0 \leq k$ (/Å) ≤ 12.7 and $1.0 \leq R$ (Å) ≤ 3.0 (MoO_3); $2.0 \leq k$ (/Å) ≤ 12.7 and $1.0 \leq R$ (Å) ≤ 3.0 ($MoCl_5$). A reasonable range of EXAFS fitting parameters: $0.700 < S_0^2 < 1.000$; $CN > 0$; $\sigma^2 > 0$ Å^2; $|\Delta E_0| < 10$ eV; R factor < 0.02.

Supplementary Table 4. The free energy changes. The free energy changes of four elementary steps for OER in MoO_3@CoO/CC, and CoO/CC systems when the applied potential is 0 V or 1.23 V, and the unit of free energy is eV.

Sample	U	step1	step 2	step3	step4
CoO/CC	0	1.65	0.72	1.90	0.65
	1.23	0.42	-0.51	0.67	-0.58
MoO_3@CoO/CC	0	0.91	1.24	1.65	1.12
	1.23	-0.32	0.01	0.412	-0.11

Supplementary Table 5. Performance comparison under different conditions. Performance comparison of MoO_3@CoO/CC under different medium conditions.

Electrolytic	10 mA cm^{-2} (Overpotential mV)	100 mA cm^{-2} (Overpotential mV)	400 mA cm^{-2} (Overpotential mV)	600 mA cm^{-2} (Overpotential mV)	800 mA cm^{-2} (Overpotential mV)
1 M KOH+1 M NaCl	298	399	505	559	611
1 M KOH+1.5 M NaCl	275	391	498	555	607
1 M KOH+ Real Seawater	297	404	531	599	664
Real Seawater	318	418	552	627	696
6 M KOH+1.5 M NaCl (80℃)	345	392	445	472	488

Supplementary Table 6. Comparison of stability of continuous oxygen generation with literature. Performance comparison of non-noble metal OER electrocatalysts in alkaline real seawater.

Material	Long-term stability	Ref.
**MoO_3@CoO/CC**	**1000 h@η_{600}**	**This work**
CoP_x@FeOOH	80 h@η_{100}	S35
$Fe_{0.01}$&Mo-NiO	50 h@η_{100}	S36
NiFe-CuCo LDH	50 h@η_{100}	S37
NiFeS/NF	24 h@η_{100}	S38
Fe(Cr)OOH/Fe_3O_4/NF	100 h@η_{100}	S39
MnCo/NiSe	200 h@η_{500}	S40
B-$MnFe_2O_4$@MFOC	100 h@η_{100}	S41
S-(Ni,Fe)OOH	100 h@η_{100}	S42
$Ni(OH)_2$-TCNQ/GP	80 h@η_{100}	S43
Ni-doped FeOOH	80 h@η_{100}	S44
0.5Fe-$NiCo_2O_4$@CC	20 h@$\eta_{21.7}$	S45
N-CDs/NiFe-LDH/NF	20 h@η_{100}	S46
NiPS/NF	24 h@η_{100}	S47
$FeOOH_{0.60}$/$Ni(HCO_3)_2$	96 h@η_{100}	S48
NiCoHPi@Ni_3N/NF	120 h@η_{100}	S49

Supplementary Table 7. Comparison of hydrogen production rate with literature. Comparison of hydrogen production rates in various seawater.

Material	Medium	H_2 (mL cm^{-2} h^{-1})	Ref.
**MoO_3@CoO/CC‖Pt/C/CC**	**Real seawater (25 °C)**	**419.4**	**This Work**
Cr_2O_3–CoO_x‖Cr_2O_3–CoO_x	Alkalized Seawater (60 °C)	300	*Nat. Energy*, **8**, 264 (2023)
Ni-NiO-Cr_2O_3‖Ni-NiO-Cr_2O_3	1 M KOH + 0.5 M NaCl	200	*PNAS*, **116**, 6624 (2019)
S-(Ni,Fe)OOH ‖ NiMoN	1 M KOH + seawater	48	*Energy Environ. Sci.* **13**, 3439 (2020)
Ir–C ‖ Pt–C	Real Seawater	1.8	*Adv. Energy Mater.* **8**, 1801926 (2018)
Pt ‖ SS	Seawater	3.3	*PNAS, 2011,* **108**, 16176 (2011).
NiCoN|Ni_xP|NiCoN‖NiCoN|NixP|NiCoN	Real Seawater	4.5	*ACS Energy Lett.* **5**, 2681 (2020)
$Ni_xB/B_4C/B\text{-}C_{PR}$/NF-B5P1 ‖ Pt/C/NF	Alkaline seawater	1.0	*ChemSusChem* **14**, 5499-5507 (2021)
Ni-doped FeOOH ‖ Pt/C	Alkaline seawater	1.2	*J. Mater. Chem. A* **9**, *9586-9592 (2021).*
NiCo@C/MXene/CF ‖ Pt/C	Seawater	255.1	*Nat.Commun.***12**, *4182 (2021).*
Ru-CoO_x/NF || Ru-CoO_x/NF	Seawater	19.8	*Small* **17**, *e2102777 (2021).*

References

[S1] Ravel, B. Newville, M.ATHENA, ARTEMIS, HEPHAESTUS: data analysis for X-ray absorption spectroscopy using IFEFFIT, *J. Synchrotron Radiat.* **12**, 537–541 (2005).

[S2] Funke, H. et al., Wavelet analysis of extended X-ray absorption fine structure data. *Phys. Rev. B* **71**, 094110 (2005).

[S3] Zabinsky, S. I. et al., Multiple-Scattering Calculations of X-Ray-Absorption Spectra. *Phys. Rev. B* **52**, 2995−3009 (1995).

[S4] Kresse, G. Furthmüller, J. Efficiency of Ab-Initio Total Energy Calculations for Metals and Semiconductors Using a Plane-Wave Basis Set. *Comput. Mater. Sci.* **6**, 15−50 (1996).

[S5] Kresse, G. Furthmüller, J. Efficient Iterative Schemes for Ab Initio Total-Energy Calculations Using a Plane-Wave Basis Set. *Phys. Rev. B* **54**, 11169−11186 (1996).

[S6] Perdew, J. P. et al., Generalized Gradient Approximation Made Simple. *Phys. Rev. Lett.* **77**, 3865−3868 (1996).

[S7] Kresse, G. Joubert, D. From Ultrasoft Pseudopotentials to the Projector Augmented-Wave Method. *Phys. Rev. B* **59**, 1758-1775 (1999).

[S8] Blöchl, P. E. Projector Augmented-Wave Method. *Phys. Rev. B* **50**, 17953−17979 (1994).

[S9] Xu, K. et al., Fluorine-induced dual defects in cobalt phosphide nanosheets enhance hydrogen eolution reaction activity. *ACS Materials Lett.* **2**, 736-743 (2020).

[S10] Tian, Y. H. et al., Engineering Crystallinity and Oxygen Vacancies of Co(II) Oxide Nanosheets for High Performance and Robust Rechargeable Zn–Air Batteries. *Adv. Funct. Mater.* **31**, 2101239 (2021).

[S11] Huang,Y. J. et al., Plasma-induced Mo-doped Co_3O_4 with enriched oxygen vacancies for electrocatalytic oxygen evolution in water splitting. *Carbon Energy*, **5**, 279 (2023).

[S12] Hu, Y. et al., Understanding the sulphur-oxygen exchange process of metal sulphides prior to oxygen evolution reaction. *Nat. Commun.* **14**, 1949 (2023).

[S13] Chen, J. S. et al., Co–Fe–Cr (oxy)Hydroxides as Efficient Oxygen Evolution Reaction Catalysts. *Adv. Energy Mater.* **11**, 2003412 (2021).

[S14] Zhang, L. L. et al., Tuning Electrical Conductance in Bilayer MoS_2 through Defect-Mediated Interlayer Chemical Bonding. ACS Nano **14**, 10265–10275 (2020).

[S15] Esmaeilirad, M. et al., Imidazolium-functionalized Mo_3P nanoparticles with an ionomer coating for electrocatalytic reduction of CO_2 to propane. Nat. Energy 8, 891–900 (2023).

[S16] Cheng, W. R. et al., Lattice-strained metal–organic-framework arrays for bifunctional oxygen electrocatalysis. *Nat. Energy* **4**, 115-122 (2019)

[S17] Zhang, Y. et al., Rapid Synthesis of Cobalt Nitride Nanowires: Highly Efficient and Low-Cost Catalysts for Oxygen Evolution. *Angew. Chem., Int. Ed.* **55**, 8670 (2016).

[S18] Ye, S. H. et al., Deeply self-reconstructing $CoFe(H_3O)(PO_4)_2$ to low-crystalline $Fe_{0.5}Co_{0.5}OOH$ with Fe^{3+}-O-Fe^{3+} motifs for oxygen evolution reaction. *Appl. Catal. B: Environ.* **304**, 120986 (2022).
[S19] Guo, D. et al., Strategic Atomic Layer Deposition and Electrospinning of Cobalt Sulfide/Nitride Composite as Efficient Bifunctional Electrocatalysts for Overall Water Splitting. *Small* **16**, 2002432 (2020).
[S20] Cui, B. H. et al., Heterogeneous lamellar-edged Fe-$Ni(OH)_2$/Ni_3S_2 nanoarray for efficient and stable seawater oxidation. *Nano Res.* **14**, 1149-1155 (2020).
[S21] Bigiani, L. et al., Selective anodes for seawater splitting via functionalization of manganese oxides by a plasma-assisted process. *Appl. Catal. B: Environ.* **284**, 119684 (2021).
[S22] Li, L. et al., Fe_2O_3/NiO interface for the electrochemical oxygen evolution in seawater and domestic sewage. *ACS Appl. Mater. Interfaces* **13**, 37152-37161 (2021).
[S23] Zhuang, L. Z. et al., Structural buffer egineering on metal oxide for long-term stable seawater splitting. *Adv. Funct. Mater.* **32**, 2201127 (2022).
[S24] Zhang, L. et al., $Ni(OH)_2$ nanoparticles encapsulated in conductive nanowire array for high-performance alkaline seawater oxidation. *Nano Res.* **15**, 6084-6090 (2022).
[S25] Ding, P. et al., N-doped carbon dots coupled NiFe-LDH hybrids for robust electrocatalytic alkaline water and seawater oxidation. *Nano Res.* **15**, 7063-7070 (2022).
[S26] Park, Y. S. et al., Ternary layered double hydroxide oxygen evolution reaction electrocatalyst for anion exchange membrane alkaline seawater electrolysis. *J. Energy Chem.* **75**, 127-134 (2022).
[S27] Tan, L. et al., Partial sulfidation strategy to NiFe-LDH@$FeNi_2S_4$ heterostructure enablehigh-performance water/seawater oxidation. *Adv. Funct. Mater.* **32**, 2200951 (2022).
[S28] Wen, N. et al., Large-scale synthesis of spinel $Ni_xMn_{3-x}O_4$ solid solution immobilized with iridium single atoms for efficient alkaline seawater electrolysis. *Adv. Sci.* **9**, 2200529 (2022).
[S29] Yang, J. et al., Quench-induced surface engineering boosts alkaline freshwater and seawater oxygen evolution reaction of porous $NiCo_2O_4$ nanowires. *Small* **18**, 2106187 (2022).
[S30]Luo, X. et al., Spherical Ni_3S_2/Fe-NiPx magic cube with ultrahigh water/seawater oxidation efficiency. *Adv. Sci.* **9**, 2104846 (2022).
[S31]Wang, X. K. et al., Asymmetric Co-N_3P_1 trifunctional catalyst with tailored electronic structures enabling boosted activities and corrosion resistance in an uninterrupted seawater splitting system. *Adv. Mater.* **34**, 2204021 (2022).
[S32] Li, J. L. et al., Nickel boride/boron carbide particles embedded in boron-doped phenolic resin-derived carbon coating on nickel foam for oxygen evolution catalysis in water and seawater splitting. *ChemSusChem* **14**, 1-10 (2021).
[S33] Wu, L. B. et al., Heterogeneous bimetallic phosphide Ni_2P-Fe_2P as an efficient bifunctional catalyst for water/seawater splitting. *Adv. Funct. Mater.* **31**, 2006484

(2021).
[S34] Zhao, Y. Q. et al., Charge state manipulation of cobalt selenide catalyst for overall seawater electrolysis. *Adv. Energy Mater*. **8**, 1801926 (2018).
[S35] Wu, L. B. et al., Rational design of core-shell-structured CoP@FeOOH for efficient seawater electrolysis. *Appl. Catal. B: Environ.* **294**, 120256 (2021).
[S36] Ning, M. H. et al., Boosting efficient alkaline fresh water and seawater electrolysis via electrochemical reconstruction. *Energy Environ. Sci.* **15**, 3945 (2022).
[S37] Yu, L. et al., High-performance seawater oxidation by a homogeneous multimetallic layered double hydroxide electrocatalyst. *PANS* **119**, e2202382119 (2022).
[S38] Chen, J. et al., High-efficiency overall alkaline seawater splitting: using a nickel–iron sulfide nanosheet array as a bifunctional electrocatalyst. *J. Mater. Chem. A* **11**, 1116 (2023).
[S39] Li, L. et al., Yang, Constructing the Fe/Cr double (oxy)hydroxides on Fe_3O_4 for boosting the electrochemical oxygen evolution in alkaline seawater and domestic sewage. *Appl. Catal. B: Environ.* **302**, 120847 (2022).
[S40] Andaveh, R. et al., Boosting the electrocatalytic activity of NiSe by introducing MnCo as an efficient heterostructured electrocatalyst for large-current-density alkaline seawater splitting. *Appl. Catal. B: Environ.* 325, 122355 (2023).
[S41] Chen, M. et al., Tuning octahedron sites in $MnFe_2O_4$ spinel by boron doping for highly efficient seawater splitting. *Appl. Catal. B: Environ.* 330, 122577 (2023).
[S42] Yu, L. et al., Ultrafast room-temperature synthesis of porous S-doped Ni/Fe (oxy)hydroxide electrodes for oxygen evolution catalysis in seawater splitting. *Energy Environ. Sci.* 13, 3439 (2020).
[S43] Zhang, L.et al., $Ni(OH)_2$ nanoparticles encapsulated in conductive nanowire array for high-performance alkaline seawater oxidation. *Nano Res.* **15**, 6084 (2022).
[S44] Park, Y. S. et al., High-performance anion exchange membrane alkaline seawater electrolysis. *J. Mater. Chem. A* **9**, 9586 (2021).
[S45] Yang, J. et al., Quench-induced surface engineering boosts alkaline freshwater and seawater oxygen evolution reaction of porous $NiCo_2O_4$ nanowires. *Small* **18**, 2106187 (2022).
[S46] Ding, P. et al., N-doped carbon dots coupled NiFe-LDH hybrids for robust electrocatalytic alkaline water and seawater oxidation. *Nano Res.* **15**, 7063 (2022).
[S47] Wang, H. Y. et al., Synergistically enhanced activity and stability of bifunctional nickel phosphide/sulfide heterointerface electrodes for direct alkaline seawater electrolysis. *J. Energy Chem.* **75**, 66 (2022).
[S48] Lin, M. et al., Engineering active sites on hierarchical transition bimetal oxyhydride/bicarbonate heterostructure for oxygen evolution catalysis in seawater splitting. *Nano Res.* **16**, 2094 (2022).
[S49] Sun, H. et al., Nickel-Cobalt Hydrogen Phosphate on Nickel Nitride Supported on Nickel Foam for Alkaline Seawater Electrolysis. *ACS Appl. Mater. Interfaces* **14**, 22061-22070 (2022).
